# COI Barcodes combined with multilocus data for representative *Aporia* taxa shed light on speciation in the high altitude Irano-Turanian mountain plateaus (Lepidoptera: Pieridae)

**DOI:** 10.1186/s12862-024-02294-3

**Published:** 2024-08-03

**Authors:** Vazrick Nazari, Vladimir Lukhtanov, Alireza Naderi, Costantino Della Bruna, Reza Zahiri, Donatella Cesaroni, Valerio Sbordoni, Valentina Todisco

**Affiliations:** 1 Department of Biology, University of Padova, Padova, Italy; 2 Department of Karyosystematics, Zoological Institute of Russian Academy of Science, St. Petersburg, Russia; 3 National Natural History Museum & Genetic Resources, Tehran, Iran; 4 Via Privata Letizia 4, Milano, 20144 Italy; 5 State Museum of Natural History Karlsruhe, Karlsruhe, Germany; 6 Department of Biology, University of Rome “Tor Vergata”, Rome, Italy; 7 Department of Environment and Biodiversity, University of Salzburg, Salzburg, Austria

**Keywords:** Sino-Himalaya, Palearctic region, Butterflies, Molecular taxonomy, Species delimitation

## Abstract

Even though the high plateaus of Qinghai-Tibet and Iran share many faunal elements, the historical biogeography of the species present in this area are not very well understood. We present a complete COI barcode library for *Aporia* Hübner and a first comprehensive phylogeny for the genus including all known species and majority of subspecies using ten available genes (COI-COII, ND1, ND5, Cytb, EF-1a, Wg, 16S, 28S-D2/D3 and 28S-D8). We then focus on *A. leucodice* (Eversmann, 1843) and related taxa in order to resolve some long-standing taxonomic issues in this species-group. Based on DNA sequence data as well as morphology, we raise *Aporia illumina* (Grum-Grshimailo 1890) stat. nov. (= *pseudoillumina* Tshikolovets 2021 syn. nov.) as a distinct species and designate a lectotype; synonymize *Aporia leucodice leucodice* Eversmann, 1843 (= *A. l. morosevitshae* Sheljuzhko, 1908 syn. nov.); and describe a new species, *Aporia ahura *sp. nov., from the Central Alborz Mountains in northern Iran.

**Supplementary Information:**

The online version contains supplementary material available at 10.1186/s12862-024-02294-3.

## Introduction

Patterns of speciation among the faunal elements shared between the high mountain plateaus of Central Asia and Iran, also known as the Irano-Turanian region [1], have been the subject of many recent studies [2–12]. This region encompasses some of the world’s most significant mountain ecosystems, including Alborz, Hindu Kush, Karakorum, Pamir, Alai, and the Himalayas. Occurrence of many high-altitude endemics in these mountains attest to the complex geological past that has shaped its current fauna. Nonetheless, the historical biogeography of this region is not very well understood.

**Fig. 1:**
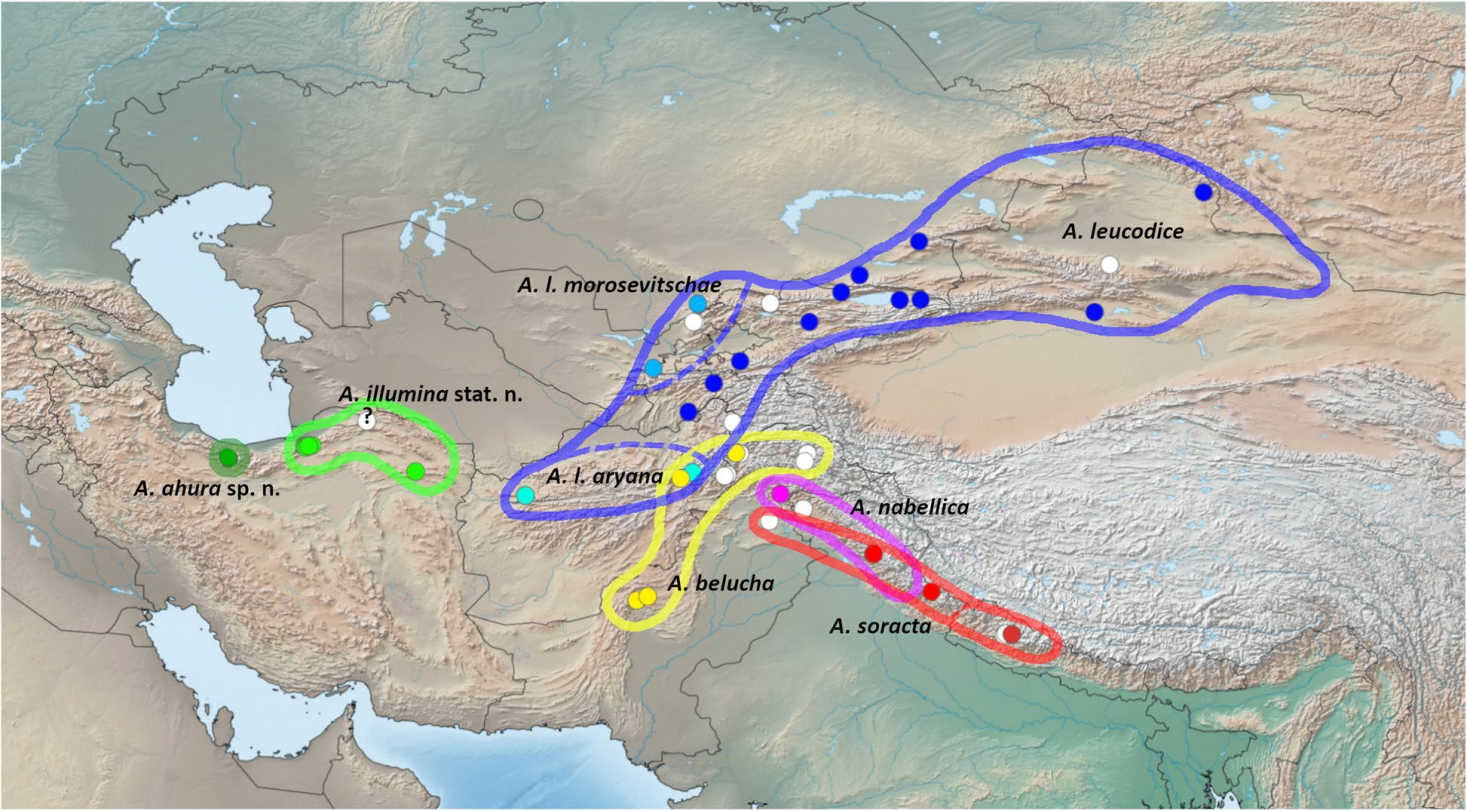
Sequenced specimens (colored) and other material (white circles) in the *A. leucodice* species group, and their approximate ranges [after 15, 20]. Map created using simplemappr.net [16]

Present in this region are a group of closely-allied, morphologically similar *Aporia* butterflies with a disputed taxonomy that occur in mid- to high elevations from the Alborz mountains in northern Iran to the Himalayas, Xinjiang in northern China, and south to Baluchistan in Pakistan: *A. leucodice* (Eversmann 1843), *A. belucha* (Marshall, 1882), *A. soracta* (Moore, 1857) and *A. nabellica* (Boisduval 1836) (Fig. 1). A fifth taxon, “*illumina*” Grum-Grshimailo, 1890 has been most recently [13] treated as a Central Asian subspecies of *A. leucodice*. Informally termed “Section *Turanoporia*” [14], these are medium-sized white butterflies with dark wing venation, characterized primarily by a synapomorphy in their male genitalia (pointed apex of uncus [15]). The closest sister species (*A. procris* (Leech, 1890)*, A. lhamo* (Oberthür, 1893)*, A. uedai* Koiwaya, 1989*, A. tsinglingica* Verity, 1911 and *A. signiana* Sugiyama, 1994) occur in South and Central China [15].

Genus *Aporia* as a whole comprises about 37 species distributed in the Palearctic region with its highest diversity in China. The type species, *A. crataegi* (Linnaeus, 1758), is widespread across the palearctic region [17]. Species radiation in *Aporia* has been the subject of several recent papers [13, 18–25], however, molecular studies so far have all had incomplete coverage [26–36]. Della Bruna et al. [15] divided the genus *Aporia* into three sections based on the shape of male uncus apex (pointed, bifid or spatulate). Deodati [29] added a forth section (intermediate), and Ding & Zhang [32] further demonstrated that a wider range of variation exists in this character and advised against recognition of sub-groups within *Aporia.*

A recent study [17] reported a deep split in the COI sequences between populations of *A. leucodice* from Central Asia and Iran, but refrained from making changes to the taxonomy of the group pending additional data. Taking advantage of this information, a subsequent paper [13] assigned the Iranian populations as a new subspecies of *A. belucha* (ssp. *pseudoillumina* Tshikolovets 2021). This arrangement, solely based on weakly-defined wing pattern elements, ignored several key points: a) the multitude of morphological characters that unite the Iranian populations with *A. leucodice* but not with *A. belucha*, b) that such a distribution pattern for *A. belucha* would be unique among the biological species in this region, and c) that the name *illumina* was already available for these populations pending designation of a lectotype. Interestingly, Todisco et al.’s paper [17] was cited as a confirmation of this new taxonomic scheme, however no specimens of *A. belucha* were sequenced or used in that study.

We began this investigation aiming to resolve the status of the Irano-Turanian taxa in the *A. leucodice* species-group using first-hand data. Subsequently, we expanded the DNA barcoding efforts to include all taxa currently recognized under genus *Aporia,* adding to our dataset also previously published data from GenBank. We thus compiled a comprehensive molecular dataset covering all recognized species and most of the subspecies in *Aporia* that allowed us to ask additional questions about this genus, and those will be addressed in subsequent publications. We then used morphological characters from wing pattern and male genitalia to further investigate the original question of species boundaries and evolution of the *A. leucodice* group in the Irano-Turanian region.

## Materials and methods

### Morphological methods

Hundreds of specimens from the private collections of the authors and collaborators, as well as published photographs of specimens from the entire range of *Aporia* were examined. Representative samples were selected for DNA barcoding and dissection (Supplementary Table S1). Male genitalia dissections were carried out by DB, VL, and Helen Alipanah (Tehran, Iran) using standard methodology. Seven syntypes of *illumina* Grum-Grshimailo were located in the collection of Zoological Institute, St. Petersburg (ZIN-RAS) and examined (Supplementary Information S2). In addition, in order to clarify the affinity of an unverified record of *A. leucodice* from western Afghanistan [37], photos of this specimen were examined courtesy of Emilio Balletto (Turin, Italy).

### Molecular methods

A total of 194 specimens representing all species and many subspecies of *Aporia* were sampled, of which 158 produced usable COI sequences (3’: *n* = 69, 5’: *n* = 157). Extraction of total genomic DNA from leg tissue, amplification and sequencing were performed in part at the University of Tor Vergata (Rome, Italy) and in part at the Centre for Biodiversity Genomics (Guelph, Ontario, Canada) using previously described protocols [29, 38]. In addition, all available sequences of *Aporia* as well as 13 outgroups pertaining to 10 genes (COI-COII, ND1, ND5, Cytb, EF-1a, Wg, 16S, 28S-D2/D3 and 28S-D8) were downloaded from GenBank and assembled into a dataset with the final length of 7,590 basepairs (Supplementary Information S2). After careful data curation, suspicious GenBank sequences were flagged and excluded from the analyses, and new sequences were deposited in GenBank (accessions PP727889– PP728045). The voucher data and accession numbers are publicly available through the BOLD dataset “DS-APLEU”, accessible at 10.5883/DS-APLEU.

Maximum Likelihood (ML) trees were generated with IQ-TREE [39] and IQ-TREE2 [40] using default parameters and 1000 ultrafast bootstrap (UFBoot) replicates. To reduce the risk of overestimating branch supports in UFBoot2 test, we implemented the –bnni option, which optimizes each bootstrap tree using a hill-climbing nearest neighbour interchange (NNI) search. To calibrate our phylogeny, two previously-published dates for this group were selected and defined as MRCA priors with normal distribution: The split between *Archonias* Hübner and *Catasticta* Butler at 4.94 mya, and the split between *Leodonta* Butler and *Pereute* Herrich-Schäffer at 6.87 mya [41]. BEAST analysis of the combined dataset was allowed to run for 20 million generation and was repeated multiple times to check for convergence and stationarity. Consensus trees obtained by TreeAnnotator v2.7.3 [42] were edited using FIGTREE 1. 4.4 [43]. Genetic distances were calculated using p-distances and Kimura-2 parameter models in MEGA 11.0.8 [44], which yielded identical results (Table 1).

**Table 1:** Kimura-2 parameter distances of COI barcodes between species in *A. leucodice* group

	*A. belucha belucha*	*A. belucha leechi*	*A. nabellica*	*A. soracta sara*	*A. soracta soracta*	***A. ahura* sp. nov**	*A. illumina illumina*	*A. leucodice aryana*	*A. leucodice leucodice*
*A. belucha belucha* (*n* = 3)	0.0 ± 0.0								
*A. belucha leechi* (*n* = 3)	2.2 ± 0.1	0.0 ± 0.0							
*A. nabellica* (*n* = 2)	5.6 ± 0.2	5.2 ± 0.0	0.0 ± 0.0						
*A. soracta sara* (*n* = 3)	5.5 ± 0.2	5.1 ± 0.0	3.3 ± 0.0	0.0 ± 0.0					
*A. soracta soracta* (*n* = 3)	6.0 ± 0.2	5.4 ± 0.1	4.0 ± 0.1	0.6 ± 0.2	0.6 ± 0.4				
***A. ahura* sp. nov.** (*n* = 4)	6.0 ± 0.5	5.8 ± 0.6	6.9 ± 0.7	7.3 ± 0.5	7.6 ± 0.5	0.6 ± 0.3			
*A. illumina* (*n* = 12)	4.9 ± 0.0	4.6 ± 0.0	6.0 ± 0.0	6.2 ± 0.0	6.5 ± 0.1	2.2 ± 1.0	0.0 ± 0.0		
*A. leucodice aryana* (*n* = 2)	6.0 ± 0.1	5.5 ± 0.1	6.6 ± 0.1	6.8 ± 0.1	6.8 ± 0.1	4.2 ± 0.9	2.6 ± 0.1	0.2 ± 0.0	
*A. leucodice leucodice* (*n* = 18)	6.2 ± 0.2	5.5 ± 0.2	7.1 ± 0.3	7.2 ± 0.2	7.2 ± 0.2	3.8 ± 0.9	2.2 ± 0.1	0.8 ± 0.1	0.1 ± 0.1

We performed molecular species delimitation (SD) analyses using two different methods. First, we implemented the Assemble Species by Automatic Partitioning (ASAP) model of Puillandre et al. [45], a distance-based method developed on the previously popular Automatic Barcode Gap Discovery (ABGD) method [46]. It analyses an alignment (usually barcode-based) and identifies the best species partitions based on genetic distances using three independent models (Jukes-Cantor, Kimura-2 parameter and simple p-distances, as in ABGD). It calculates species threshold scores, and the lowest score is the best species partition scheme. ASAP analyses were carried out on webserver https://bioinfo.mnhn.fr/abi/public/asap/ using default settings and a K80 model. In addition, we performed a Bayesian implementation of the Poisson-tree-process (PTP) model, a tree-based method derived from Zhang et al. [47], which was applied on the concatenated ML tree resulting from the IQ-TREE2 analysis of the multi-locus dataset. PTP analysis was carried out on the PTP webserver available at https://species.h-its.org/ with default parameters [47] (Supplementary Information S2). In all of these analyses, the total number of sequences was 315, representing about 37 morphological species and 13 outgroup taxa.

### Biogeographic analysis

Eight distribution areas for the range of *Aporia* were selected: Mountains of SW China, Tibetan plateau, Eastern China and Japan, Himalayas, India and SE Asia, N China/Central Asia to Hindukush, the Iranian Plateau, and the rest of the Palearctic region. We used the R package BioGeoBEARS [48] to reconstruct the biogeographic history of *Aporia*. The program compares three possible models of past geographical range estimation based on the Akaike information criterion and, for each of them, also a variant with a founder effect (parameter j): dispersal– extinction–cladogenesis (DEC), dispersal–vicariance analysis (DIVALIKE) and BI for discrete areas (BAYAREALIKE). The program then reflects these likelihoods as pie charts with all possible ranges for each node and their respective probabilities. We allowed for a maximum of four possible ancestral areas.

## Results

### Morphological results

**Fig. 2:**
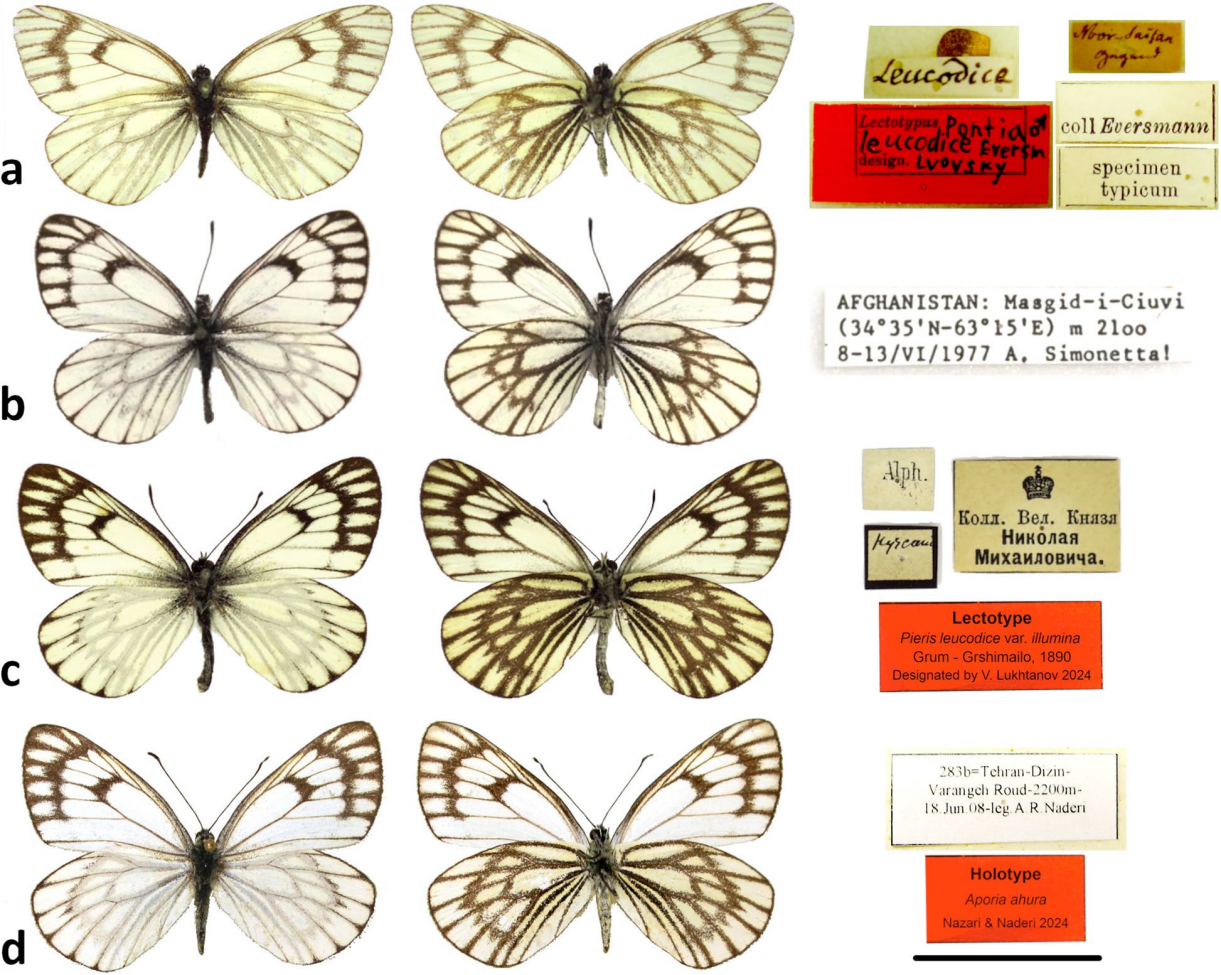
**a** *A. leucodice* Lectotype (ZIN-RAS), **b** *A. leucodice aryana* from W Afghanistan (Photo: E. Balletto), **c** *A. illumina* Lectotype (ZIN-RAS), here designated;** d** *A. ahura* **sp. n.**, Holotype. Scale bar is 2 cm

A previously unverified record of *A. leucodice* from Western Afghanistan (Herat: Masgid-i-Chiuvi, 34°35'N, 63°16'E, 2100 m, 8–13.vi.1977; c.f. [37]) was determined to belong to ssp. *aryana.* The known range of this subspecies therefore extends from Eastern (Panjshir) to Western Afghanistan (Herat). The UNH pattern of this subspecies is characteristic of *A. leucodice* (Fig. 2a), and genetically it appears on average 0.8% barcode divergent from *A. leucodice* (Fig. 2b, Table 1).

**Fig. 3:**
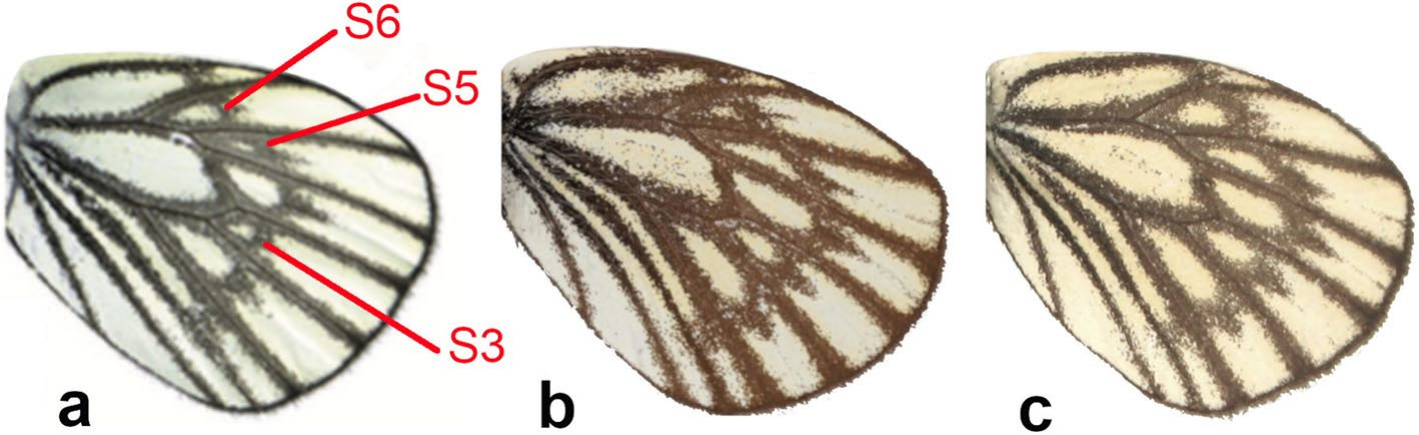
Putative diagnostic characters on the underside of the hindwings in **a** *A. leucodice* (Kazakhstan)*,* **b** *A. illumina* (Shahkuh, NE Iran) and **c** *A. ahura* sp. n. (Dizin, N Iran)

**Fig. 4:**
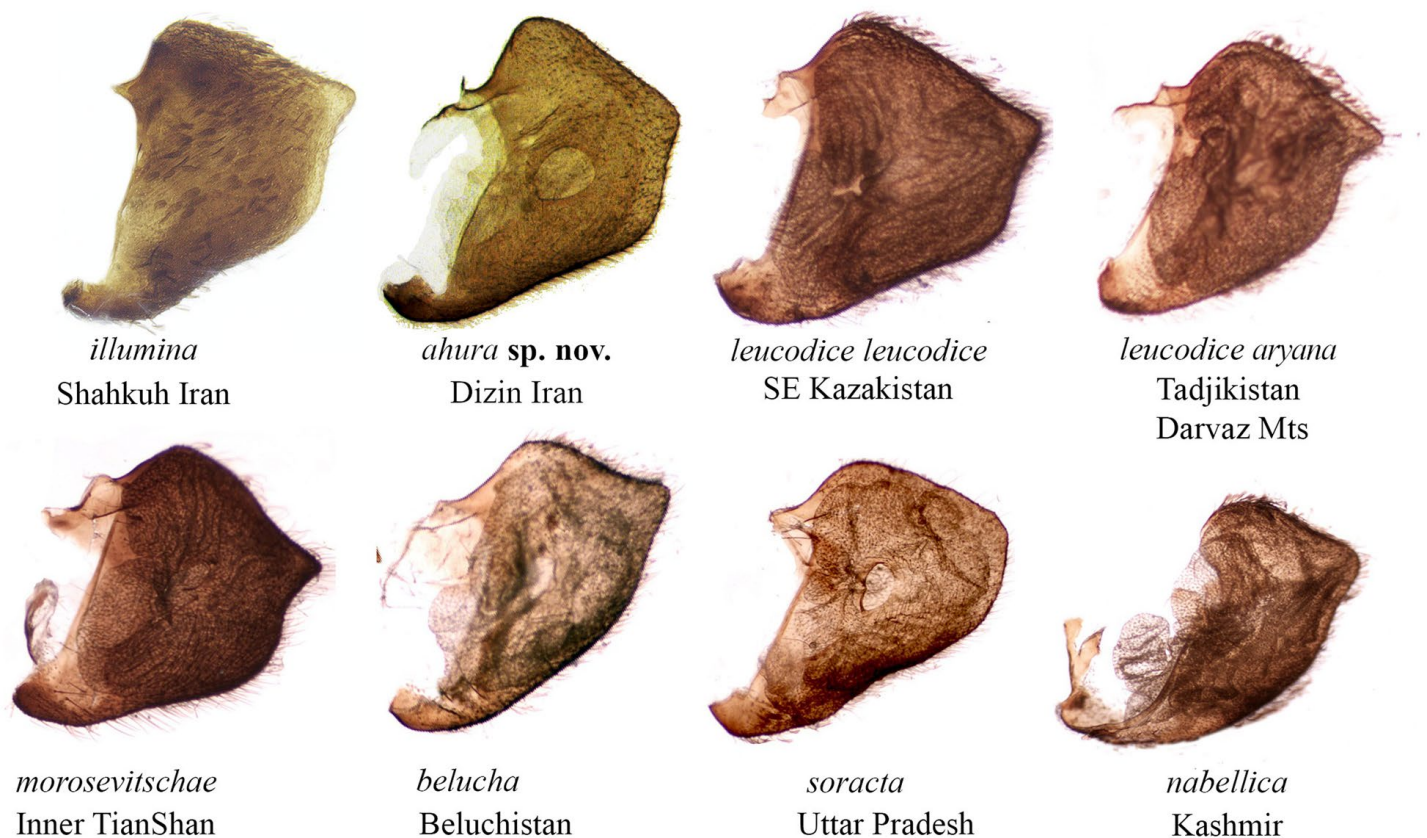
Left valve in male genitalia of the species in *Aporia leucodice* species-group (specimens in collection of VL, AN, and CDB)

We note putative diagnostic characters on the underside of the hindwings (UNH) of the adult butterflies in the *A. leucodice* group, namely the development (size) of the patches in spaces S3, S5 and S6 (Fig. 3). In addition, our male genitalia dissections of taxa in the *A. leucodice* species complex showed clear differences in the shape of the valve between the examined taxa (Fig. 4). We did not investigate the morphological characters in other species-groups in this study.

### Molecular results

**Fig. 5:**
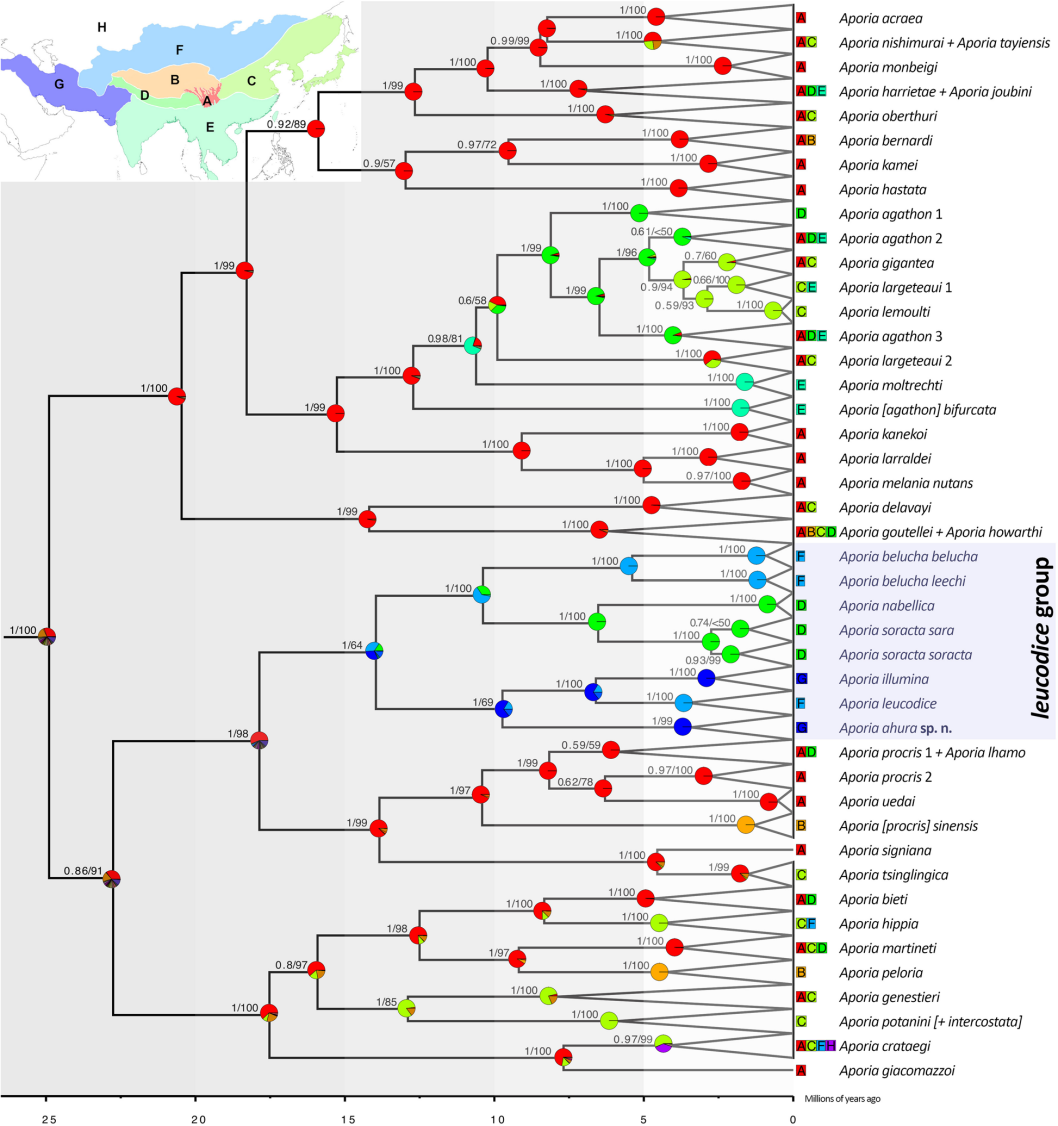
Multigene BEAST phylogeny of *Aporia.* Values above nodes are Bayesian Posterior Probabilities followed by Bootstrap for 1000 replicates

Maximum Likelihood and Bayesian analyses produced similar tree topologies with well-supported deeper nodes (Fig. 5, Supplementary Information S2). For *A. giacomazzoi* Della Bruna et al. 2003, we obtained only a partial DNA barcode fragment which showed unstable position throughout the phylogenetic analysis. In addition, our single specimen of *A. howarthi* Bernardi, 1961, despite morphologically being related to the *A. crataegi* species-group, appeared within the *A. goutellei* (Oberthür, 1886) clade. Since in both cases single specimens are involved, we consider those as *incertae sedis* until further sequencing confirms their status.

We noticed many misidentified or contaminated samples in GenBank from previous studies (Table 2). These were either excluded from our analyses or used with corrected identifications. In addition, we discovered that a previously published sequence of *A. illumina* as being from Kopet-Dagh mountains in NE Iran (MN993017) actually belonged to a mislabeled specimen originating from Central Alborz Mountains in Northern Iran.

**Table 2:** Problematic sequences in GenBank that were either excluded or renamed in this study

Original ID	Corrected ID	Sample ID	Issue	Accession(s)	Locality	Reference
*Aporia hippia*	*Aporia genestieri*	[not given]	misidentified	NC_033889	China: Gansu, Diebu County, Henzu	Cao, Y. et al. 2016 [34]
*Aporia bieti*	*Aporia hippia*	[not given]	misidentified	NC_033888	China: Gansu, Diebu County, Henzu	Cao, Y. et al. 2016 [34]
*Aporia agathon*	*Aporia agathon*	A.aga1	odd sequences	KM669578, KM669305, KM669358, KM669413, KM669525	China: Tibet, Motuo	Ding, C. 2016 [33]
*Aporia delavayi*	*Aporia delavayi*	A.del1	odd sequences	KM669361, KM669526	China: Shaanxi, Huoditang	Ding, C. 2016 [33]
*Aporia genestieri*	*Aporia genestieri*	A.gen1	odd sequences	KU921259, KU921253	China: Shaanxi, Hanzhong	Ding, C. 2016 [32, 33]
*Aporia gigantea*	*Aporia largeteaui*	A.gig1	misidentified	KU921260, KU921254	China: Sichuan, Mt. Emeshan	Ding, C. 2016 [32, 33]
*Aporia martineti*	*Aporia martineti*	A.mar1	contminated	KU921265	China: Qinghai, Datong	Ding, C. 2016 [32, 33]
*Aporia oberthuri*	*Aporia oberthuri*	A.obe1	odd sequences	KM669527	China: Shaanxi, Huoditang	Ding, C. 2016 [33]
*Aporia procris*	*Aporia procris*	A.pro1	odd sequences	KM669582, KM669310, KM669363, KM669632, KM669417, KM669528, KM669473	China: Shaanxi, Taochuan	Ding, C. 2016 [33]
*Aporia acraea*	*Aporia goutellei*	APA	misidentified	ON216403, ON216497, ON216585	China: Sichuan, Yaan, Baoxing Country, Mt. Jiajinshan	Ge,S.X. et al. 2023 [49]
*Aporia goutellei*	*Aporia oberthuri*	APG	misidentified	ON216320, ON216404, ON216498, ON216586	China and adjacent regions	Ge,S.X. et al. 2023 [49]
*Aporia goutellei*	*Aporia acraea*	Aporia_goutellei	misidentified	ON533767	China: Sichuan, Yaan, Baoxing Country, Mt. Jiajinshan	Ge,S.X. et al. 2023 [49]
*Aporia intercostata*	*Aporia gigantea*	HJS-200602	contminated	EF584851, EF584874	China: Anhui, Huangshan	Xu,L. and Hao,J. unpublished
*Aporia potanini*	*Aporia crataegi*	HJS-200603	odd sequences	EF584852, EF584875	China: Heilongjiang, Qiqihaer	Xu,L. and Hao,J. unpublished
*Archonias brassolis*	*Catasticta nimbice*	MGCL:LEP-53237	same as OK747128	OK746773	Brazil: Santa Catarina, Florianopolis, Lagoa Peri, Trilha Gurita	Kawahara et al. 2022 [41]

Results of our clock analysis corresponds well with the divergence times inferred in previous studies (e.g. [41]), with only minor deviations. Our phylogenetic analysis revealed a more or less uniform rate of diversification in *Aporia* (Fig. 5), and thus we do not recommend splitting the genus into subgeneric categories based on geological age.

Within the *A. leucodice* group, geographically well-delineated haplotypes corresponding to distinct subspecies were noted in *A. soracta* and *A. belucha*. In our phylogenetic analyses, *A. belucha* appeared as sister to *A. nabellica* and *A. soracta* and very distant from *A. leucodice*. Contrary to Tshikolovets [13], North Iranian populations were not associated with *A. belucha*, but appeared as a distinct sister to the nominotypical *A. leucodice*. Moreover, within the Iranian populations, we unexpectedly found two deeply divergent linages that were paraphyletic with respect to *A. leucodice*, with samples from Central Alborz (Dizin) that appeared sister to the others (Fig. 5). The COI barcodes of the population from Dizin showed an average 2.2% distance from those in NE Iran and 3.8–4.2% from *A. leucodice* (Table 1).

The three distance-based species-delimitation models with the highest ASAP scores yielded 42 putative species of *Aporia* (55 with the outgroups), corresponding more or less with the recognized number of morpho-species in the genus (Supplementary Information S2). Within the *A. leucodice* group, all three models delimited six putative species: *A. belucha belucha, A. b. leechi* Moore, 1904*,* the populations from NE Iran, the population from Central Alborz (Dizin)*, A. leucodice*, and *A. nabellica* (even though the latter appeared in a separate cluster). On the other hand, the tree-based PTP models over-estimated the number of putative ingroup species for genus *Aporia* as 101 by Bayesian and 85 species by Maximum Likelihood methods. The number of putative species partitions delimited for the *A. leucodice* clade inferred by PTP ML method was 17 and by PTP B method was 16 (Supplementary Information S2). Both PTP methods also identified the population from N Iran (Dizin) as a new species with high support value (0.78).

### Biogeographic analysis

In our BioGeoBears analysis, the model with the highest likelihood was DEC + J, which, in addition to the likelihood implementation of the processes assumed by DIVA, also considers sympatry and founder effect. Our results place a high probability on southwest China ecozone having played a major role in the early radiation of *Aporia*, with subsequent dispersal to other regions (Fig. 5, Supplementary Information S2). The *A. leucodice* species-group, distributed from North China to the Iranian Plateau, seems to have split around 18 million years ago from a common ancestor with the *A. procris* species-group that today occur in SW China and the Tibetan plateau.

## Discussion

### Phylogeny of the genus *Aporia*

A comprehensive and dated phylogeny for genus *Aporia* has so far been lacking. Based on its close affinity with the fossil *Coliates proserpina* Scudder 1875, Cao et al. [34] estimated the minimum age of *Aporia* crown group at 33.5–30 mya during the Oligocene, and Kawahara et al. [41] estimated the split between *A. crataegi* and *A. agathon* (Grey, 1831) at 20.25 mya. These dates are comparable with an earlier estimate suggesting that early differentiation in *Aporia* occurred over a relatively long period during the mid-Tertiary [27]. However, another study using the substitution rate of 1.15% per lineage per million years for COI [50] estimated the early diversification of Sino-Himalayan species of *Aporia* to have occurred between 10–6 mya [29]. Using two relatively shallow calibration points among the outgroups (c.f. [41]), we found the median age of *Aporia* at 24.9 million years old, more or less in line with majority of previous studies.

The results of molecular species delimitation analyses (ASAP and PTP) showed an overall tendency towards over-splitting in the latter method. Previous studies have shown a decrease in delimitation accuracy for PTP method when applied to the unevenly sampled datasets [47, 51, 52], and it appears that the sampling imbalances across taxa in our dataset has similarly resulted in an over-estimation of number of taxa.

On the contrary, the ASAP analysis identified only 42 putative species of *Aporia*, comparable to the 37–39 morpho-species recognized within this genus [15], but with some notable exceptions. While some morpho-species were lumped together (e.g. *A. nishimurai* Koiwaya, 1989 + *A. tayiensis* Yoshino, 1995*, A. signiana* + *A. tsinglingica* Verity, 1911*, A. potanini* Bernardi, 1963 + *A. intercostata* Bang-Haas, 1927 etc.), some subspecies were identified as distinct species (e.g. *A. belucha leechi*, *A. bieti lihsieni* Bang-Haas, 1933*, A. procris sinensis* (Bang-Haas, 1927) etc.). The taxonomy of certain species (e.g. *A. agathon*, *A. largetaui* (Oberthür, 1881)*, A. procris* etc.) appear to be in dire need of comprehensive revisions. Nonetheless, in all of our models, the populations of *A. leucodice* from Central Asia, NE Iran and N Iran (Dizin) were unambiguously recognized as three distinct species.

The split between the population in N and NE Iran (mean: 9.73 my) may have occurred partly due to a shift in the host-plant species. The larval hosts of taxa in *A. leucodice* group have a difficult taxonomy with rampant hybridization [53]. Genus *Berberis* L. has four species in Iran, with the common Barberry (*B. vulgaris* L.) extending from NW Iran to Central Alborz, while in the Shahkuh region and Northern Khorasan occur two other species, *B. orthobotrys* Bien. ex Aitch. and *B. integerrima* Bunge ( [54, 55]; Mozaffarian, pers. comm.). Many more species of *Berberis* are distributed in Pakistan, India, Central Asia and China [56, 57], corresponding to the high diversity of *Aporia* species found in this region.

### Taxonomic considerations

The major monophyletic clades observed in our phylogenetic analysis (Fig. 5) did not always correspond with those identified previously based on the shape of the male uncus (pointed, bifid, intermediate and spatulate) [15, 29, 32]. We found geographically well-delineated unique haplotypes within many species corresponding to subspecies or even distinct species. On the other hand, we also found lack of genetic differentiation between several taxa that are usually recognized as distinct.

*A. leucodice* (Eversmann 1843), described from Tarbagatai Mountains in Kazakhstan [58], has a wide distribution from NW China to Afghanistan (Fig. 2a). The name *morosevitshae* Sheljuzhko, 1908 was given to lighter specimens of *A. leucodice* flying in the Western Tian-Shan mountains [59]. However, light and dark specimens can often be found flying together in this region with DNA barcodes that are identical to other populations across the range of *A. leucodice*. Thus, here we propose *Aporia leucodice* (Eversmann, 1843) = *morosevitshae* Sheljuzhko, 1908 syn. nov.

The fact that Northern Iranian lineages in this group are sister to the nominotypical Central Asian *A. leucodice* with high support invalidates their association with *A. belucha* (i.e. [13]). The UNH wing pattern and the genitalia of the dissected specimens from the two populations in N and NE Iran showed clear differences from the Central Asian *A. leucodice* and all other species in this group (Figs. 3 and 4). It must be noted that a large degree of individual variation in male genitalia has been previously documented in *A. procris* [23], which also appears to correlate with its high genetic diversity. Further research is therefore needed to clarify the extent of individual variation in the male genitalia within various populations of *A. leucodice* species-group.

Taking all of the information above into account, here we assign the rank of species to the two populations in N and NE Iran and formally separate them from *A. leucodice*. The name *illumina* Grum-Grshimailo (1890) is available for one of these populations. In describing “var. *illumina*”, Grigory Grum-Grshimailo gave the following type localities: “*la forme plus foncée, habitant les pentes septentrionales du Thian-Chan, des monts Alaï et la partie septentrionale de la Perse montagneuse*” [60]. Even though Grum-Grshimailo collected *A. leucodice* in many parts of Central Asia, he himself never collect *A. leucodice* in Iran. Nevertheless, he examined Persian specimens that were held at the Zoological Institute in St. Petersburg at the time. Before 1890, only three collectors had collected this species in Iran: Joseph Haberhauer in 1867 and 1869 (c.f. [61, 62]), Hugo Christoph in 1870, 1871 and 1873 [63], and Alfred Otto Herz in 1887. They traveled through Astrabad (now Gorgan) and collected around “Hadschyabad”, “Schakuh”, “Tasch”, etc.

Joseph Haberhauer was a German collector who traded or exchanged butterflies with Russian lepidopterists N. Erschoff, S. Alpheraky and Grand Duke Nikolai Mikhailovich Romanov, either by himself or through Julius Lederer and later through Otto Staudinger. The Haberhauer samples are preserved in Berlin, but it is unlikely that these were ever seen by Grum-Grshimailo or used as “types” for *illumina*. On the other hand, Hugo Christoph and Alfred Otto Herz both lived and worked in St. Petersburg. Between 1870 and 1873, Christoph spent several weeks in Shahkuh, where he collected “*Pieris leucodice*” [63]. Christoph’s specimens were in St. Petersburg when Grum-Grshimailo wrote the description of *illumina*. These specimens do not have a collector’s name, and in accordance with the traditions of the 19th century, their labels indicate not the exact place of collection, but either the nearest large city (“Astrabad”) or the historical name of the region (“Hyrcania”). It is clear that all these butterflies come from the collections of either Haberhauer or Christoph. It is also clear that Grum-Grshimailo saw these butterflies, since he did not have his own collections from Iran. While preparing his monograph on Pamir butterflies, Grum-Grshimailo worked closely with Christoph, N. Erschoff and Grand Duke N. Romanov [60].

In the collection of the ZIN-RAS, in addition to specimens labeled “Hyrcania” or “Astrabad”, we found one female from “Schahkuh”, collected 2 June 1887 by Herz. However, we were unable to find in this collection undoubted syntypes of *illumina* from Tian-Shan or Alai. Grum-Grshimailo himself collected *A. leucodice* in various places in Central Asia and surely also examined those while describing *illumina*, but these specimens are no longer in ZIN-RAS, and it is possible that these were later transferred to London.

Even though *illumina* was described from a series of specimens from different parts of Central Asia as well as from the mountains of northern Iran, a tradition arose almost immediately to use the name *illumina* specifically for the Iranian population. Röber (in Seitz [64]) was the first reviewer who used this name solely for Iranian populations, emphasizing their morphological differences from the nominotypical *leucodice*. Following Röber, the name *illumina* was fixed in the literature specifically for the Iranian population (some recent sources are exceptions; e.g. [20, 21, 65, 66]). Here we follow recommendation 74A of the International Code of Zoological Nomenclature (ICZN)^1^ to designate a ZIN-RAS syntype from “Hyrcania” (Fig. 2a) as the lectotype, and raise the rank of *illumina* to species:

- ***Aporia illumina ***Grum-Grshimailo, 1890 stat. nov. (Fig. 2c)
- *Pieris leucodice* var. *illumina* Grum-Grshimailo 1890:15. TL: “habitant les pentes septentrionales du Thian-Chan, des monts Alaï et la partie septentrionale de la Perse montagneuse”.
-  = *Aporia belucha pseudoillumina* Tshikolovets 2021:640, TL: [Iran], Hadschyabad, Hbhr [Haberhauer], ex coll. Staudinger (ZMHB). syn. nov.
- Lectotype ♂(here designated): [white rectangular label: upperside of the label with black border, hand-written in black “Hyrcania”; underside of the label without border, type set “Alph.”[eraky collection] / [large white label] “[Image of Royal crown] / Кoлл. Beл.Князя / Hикoлaя / Mиxaилoвичa” / red rectangular label “Lectotype Pieris leucodide var. illumina Grum-Grshimailo, 1890 Designated by V. Lukhtanov 2024”. Deposited in the coll. Zoological Institute, Russian Academy of Sciences (ZIN-RAS), St. Petersburg, Russia. Designated by V. Lukhtanov.

The type locality of *Aporia illumina* stat. nov. includes the eastern Alborz mountains, i.e. Shahkuh, “Hyrcania”, “Hadschyabad” [= Gorgan], “Tasch” etc. Therefore, the population from Shahkuh appear to represent the nominotypical taxon *illumina*. Specimens from Kuh-e Sorkh in Northern Khorasan also belong to *illumina.* However, since our single specimen from Kopet-Dagh was found to be mislabelled, the genetic affiliation of the populations from Kopet-Dagh remains to be confirmed. The divergent cluster of *Aporia illumina* from Central Alborz mountains in N Iran represents populations that require a new name. Here we propose:

- ***Aporia ahura*** Nazari & Naderi sp. nov. (Fig. 2d).
- Material. Holotype: ♂, Iran, Alborz Province, Dizin, Varangehrood, 2200m, 18.VI.2008, leg. A.R. Naderi; SampleID 283b, dissection HA-2964. Not barcoded. Deposited in the coll. National Natural History Museum and Genetic Resources, Tehran, Iran.
- Paratypes (7♂♂, 5♀♀)**:** Iran, Alborz Province, same data as holotype, 4♂♂1♀coll. A.R. Naderi (SampleIDs ARPI-9999–030 to 032); 1♂ 3♀♀ leg. A.R. Naderi, coll. P. Zehzad (no SampleIDs); 1♂ leg. A.R. Naderi, coll. A.H. Harandi (no SampleIDs); 1♂2000m, 21.VI.2012, leg. A.R. Naderi, coll. P. Zehzad (no SampleIDs); 1♀ Chalus road, Dizin, 2500 m, 2.VII.1994, leg. and coll. A.R. Naderi (barcoded, SampleID ARPI-9999–029).
- Description. Male (Fig. 2d). Head black, frons white with black hairs. Antenna uniformly black, tip of the club white. Thorax black with gray hairs, abdomen black dorsally, grayish white ventrally. Legs black with white scales.
- Forewing length 20–22 mm. Dorsal side of wings white; forewing with dark basal suffusion extending from base along the inner margin, veins black, a large black discoidal spot, and a broad marginal band extending from the apex to S2 with white internal scale-shaped patches. Hindwing rounded, white with narrow black margin, veins black only at marginal 1/3 and more intensively developed towards at the edge of the wings; weakly-developed postdiscal markings in the form of small arrows pointing outwards often present in S4–S6. Fringes on both wings gray, uneven. Ventral side of wings contrasted, forewing white except the apical area yellowish within the marginal band; veins grayish at base to more intense blackish towards the margin; discoidal spot and marginal band well-developed, blackish-brown; white internal patches within the marginal band wider than dorsal side and not scale-shaped. Hindwing yellowish, veins broadly suffused with gray scales, an additional streak present along the middle of S1; a continuous postdiscal band of chevrons of similar width extending from the inner margin to the upper half of S1.
- Male genitalia (Fig. 4). Heavily sclerotized. Ring slender, straight, perpendicular to saccus and tegumen; uncus broad at base, gradually narrowed into a pointed tip; saccus short and broad. Valve nearly triangulate, with dorsal base concave and ventral margin convex, tip blunt, fovea large and dorsoventrally elongate. Aedeagus robust, evenly curved with a trochanter at its ventral base. Juxta v-shaped with two arms widely apart.
- Female: Forewing length: 22–24 mm. Similar to male but wings often ore elongate, upperside dark marginal marking paler, discoidal spot often narrower than male. Ventral side of wings similar to male, dark markings paler.
- Female genitalia: Not examined.
- Individual variation. The intensity of dark markings on both sides of the wings to some extent vary.
- Diagnosis. Similar to *A. illumina*, forewings wider (narrower and somewhat more elongate in *A. illumina*), ground color and markings generally paler and less developed, UNH patch in S6 always well developed (usually small or obscured in *illumina*); ♂genitalia valve edge smooth, without pointed tip.
- Molecular characterization. *Aporia ahura* sp. nov. shows a COI barcode distance of 2.2 ± 1.0% from *A. illumina*, differing from it by 21 fixed substitutions along the 658bp of the DNA barcode region. The available sequences for *A. ahura* sp. nov. (*n* = 4) varied in length, nevertheless they showed variation in six additional sites resulting in four different haplotypes. In contrast, all barcoded specimens of *A. illumina* (*n* = 12), even though originating from often distant localities, were barcode identical.
- Distribution and bionomics. The new species is so far found only in Central Alborz mountains, Alborz province in Northern Iran (Fig. 1). In addition to the type locality (Dizin), specimens illustrated from Marzanabad in Central Alborz [67] belong to *A. ahura.* The new species inhabits altitudes between 2000–2500 m a.s.l. in mountain slopes with thick vegetation and *Juniperus* trees. Adults fly from mid-June to early July; they have a gentle flight and can often be seen nectaring on flowers of *Berberis* and *Colutea.*
- Etymology. The species name *Ahura* (Lord) is an ancient Iranian (Avestan) designation for a particular class of Zoroastrian divinities that also includes *Ahuramazda*, the creator deity in Zoroastrianism.

### Proposed taxonomic scheme for the *Aporia leucodice* species-group based on the results of this study

1. *Aporia belucha* Marshall, 1883
  ssp. *belucha* Marshall, 1883
  ssp. *leechi* (Moore, [1904])
2. *Aporia nabellica* (Boisduval, 1836)
  ssp. *nabellica* (Boisduval, 1836)
  ? ssp. *hesba* Evans, 1912 (no molecular data available)
3. *Aporia soracta* Moore, 1857
  ssp. *soracta* Moore, 1857
  ssp. *sara* Evans, 1932
4. *Aporia leucodice* Eversmann, 1843
  ssp. *leucodice* Eversmann, 1843
  ssp. *aryana* Wyatt and Omoto, 1966
5. *Aporia illumina* Grum-Grshimailo, 1890 stat. nov.
  =*morosevitshae* Sheljuzhko, 1908 syn. nov.
6. *Aporia ahura* Nazari & Naderi sp. nov.
  =*pseudoillumina* Tshikolovets 2021 syn. nov.

### Supplementary Information

Supplementary Material 1.

Supplementary Material 2.

## Data Availability

New sequences generated in this study are deposited in GenBank (accessions PP727889– PP728045). The voucher data and accession numbers are publicly available through the BOLD dataset “DS-APLEU”, accessible at 10.5883/DS-APLEU.

## Abbreviation

ZIN-RAS: Entomological collection, Zoological Institute, St. Petersburg, Russia

## Footnotes

1 ICZN Recommendation 74A: Agreement with previous restriction. In designating a lectotype, in order to preserve stability of nomenclature an author should act consistently with, and in any event should give great weight to, previously accepted taxonomic restrictions of the application of the name [68].
