# Supplementary material for: COI Barcodes combined with multilocus data for representative Aporia taxa shed light on speciation in the high altitude Irano-Turanian mountain plateaus (Lepidoptera: Pieridae)
Source: BMC Ecol Evol. 2024 Aug 3;24:105. doi: 10.1186/s12862-024-02294-3 (PMC11297774; doi:10.1186/s12862-024-02294-3)

Nazari et al. 2024 - Supplementary Information S2

Table of contents

1. Syntypes of *Aporia illumina* in ZIN-RAS
2. Maximum Likelihood tree obtained from IQTree analysis
3. Results of the ASAP analysis (JC, K2P and p-distances)
4. Results of the PTP-ML and PTP-BI analysis
5. Results of BioGeoBEARS analysis (DEC+J model)

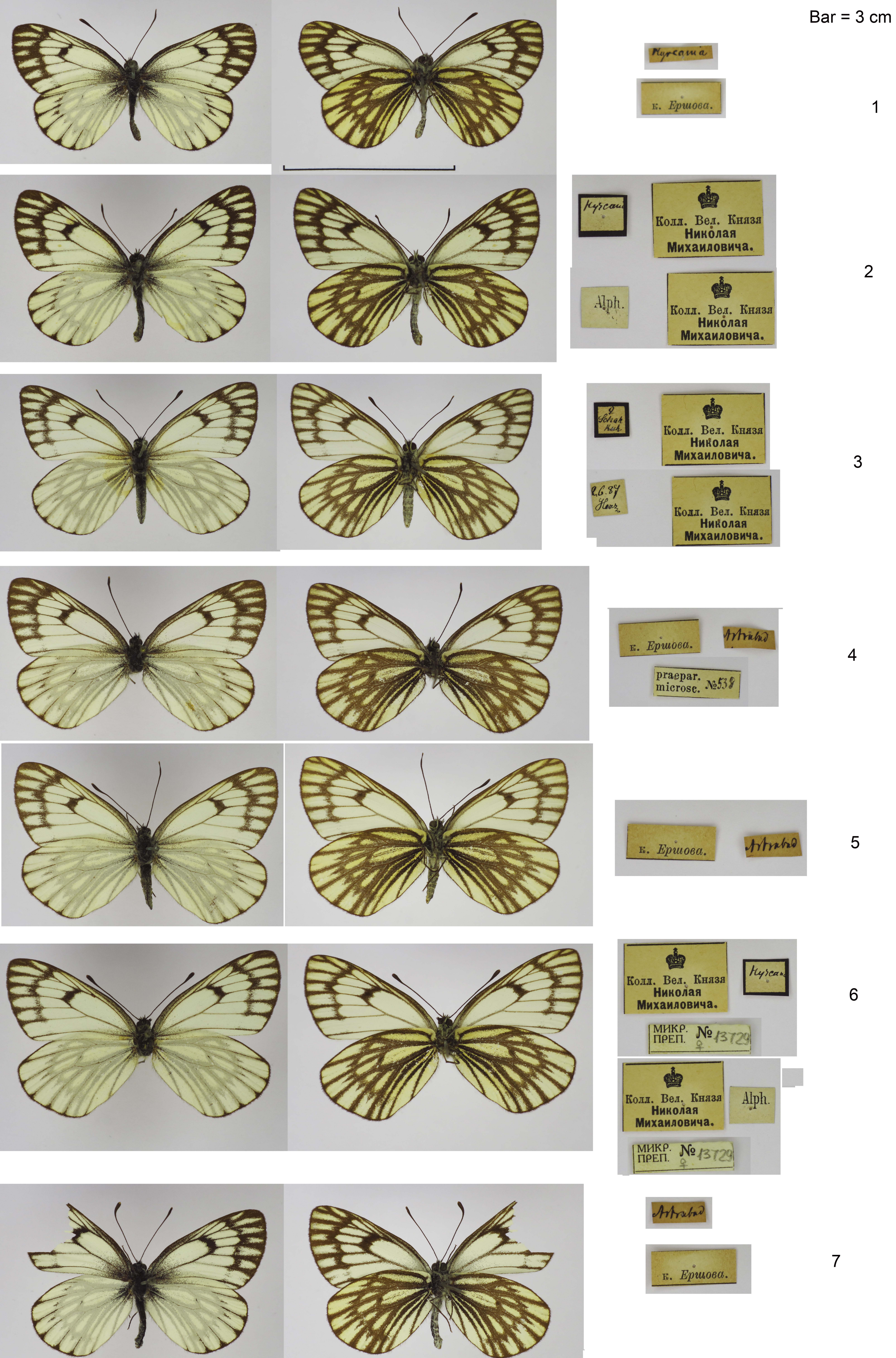
Bar = 3 cm
1
к. Ершова.
2
Колл. Вел. Князя Николая Михаиловича.
Alph.
Колл. Вел. Князя Николая Михаиловича.
3
Колл. Вел. Князя Николая Михаиловича.
Колл. Вел. Князя Николая Михаиловича.
4
к. Ершова.
praepar. microsc. №538
5
к. Ершова.
6
Колл. Вел. Князя Николая Михаиловича.
МИКР. ПРЕП. № 13729 ♀
Колл. Вел. Князя Николая Михаиловича.
Alph.
МИКР. ПРЕП. № 13729 ♀
7
к. Ершова.

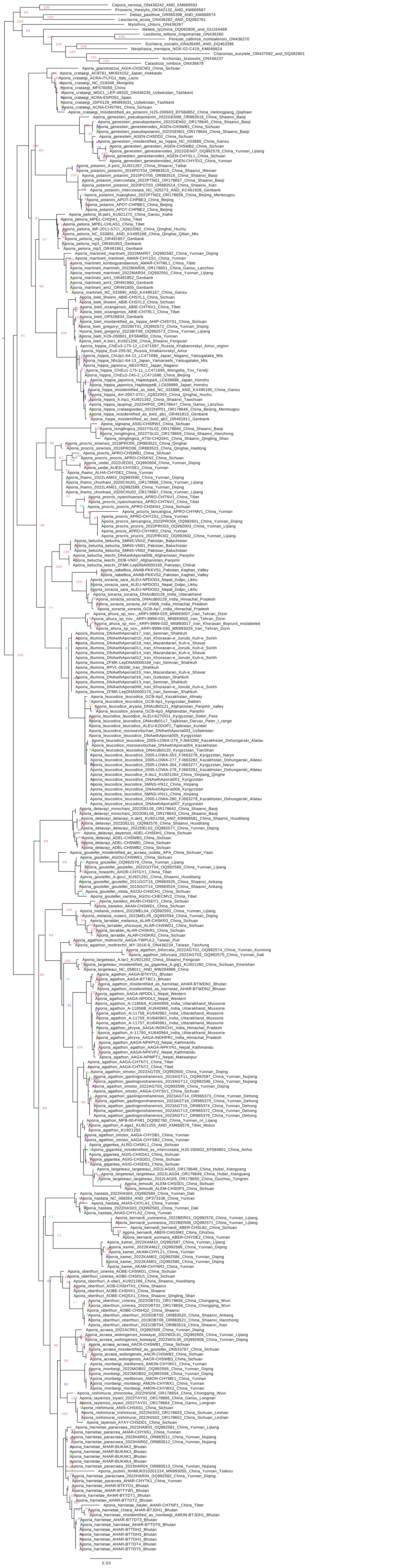

Cepora_nerissa_ON436242_AND_KM669593
Prioneris_thestylis_OK342132_AND_KM669587
Delias_pasithoe_OR965398_AND_KM669574
Leuciacria_acuta_ON436262_AND_DQ082761
Mylothris_chloris_ON436267
Melete_lycimnia_DQ082800_and_GU164469
Leodonta_tellane_tingomariae_ON436260
Pereute_callinice_numbalensis_ON436270
Eucheira_socialis_ON436495_AND_DQ463396
Neophasia_menapia_NGK-02-C419_KM046824
Charonias_eurytele_ON437092_and_DQ082801
Archonias_brassolis_ON436237
Catasticta_nimbice_ON436678
Aporia_giacomazzoi_AGIA-CHSCW3_China_Sichuan
Aporia_crataegi_AC8761_MK924152_Japan_Hokkaido
Aporia_crataegi_ACRA-ITLFG1_Italy_Lazio
Aporia_crataegi_NC_018346_Mongolia
Aporia_crataegi_MF576059_China
Aporia_crataegi_MGCL_LEP-48320_ON436235_Uzbekistan_Tashkent
Aporia_crataegi_ACRA-ESPDS1_Spain
Aporia_crataegi_JDF0129_MN993031_Uzbekistan_Tashkent
Aporia_crataegi_ACRA-CHSTM1_China_Sichuan
Aporia_crataegi_misidentified_as_potanini_HJS-200603_EF584852_China_Heilongjiang_Qiqihaer
Aporia_genestieri_pseudopotanini_2022GEN08_OR883518_China_Shaanxi_Baoji
Aporia_genestieri_pseudopotanini_2022GEN02_OR178645_China_Shaanxi_Baoji
Aporia_genestieri_genestieroides_AGEN-CHSWB1_China_Sichuan
Aporia_genestieri_pseudopotanini_2022GEN01_OR178644_China_Shaanxi_Baoji
Aporia_genestieri_AGEN-CHSDD2_China_Sichuan
Aporia_genestieri_misidentified_as_hippia_NC_033889_China_Gansu
Aporia_genestieri_genestieroides_AGEN-CHSWB2_China_Sichuan
Aporia_genestieri_genestieroides_2022GEN07_OQ992578_China_Yunnan_Lijiang
Aporia_genestieri_genestieroides_AGEN-CHSYL1_China_Sichuan
Aporia_genestieri_genestieroides_AGEN-CHSYV2_China_Yunnan
Aporia_potanini_A.pot1_KU921267_China_Shaanxi_Taibai
Aporia_potanini_potanini_2016POT04_OR883515_China_Shaanxi_Weinan
Aporia_potanini_potanini_2015POT05_OR883516_China_Shaanxi_Baoji
Aporia_potanini_intercostata_2022PTN01_OR178657_China_Shaanxi_Baoji
Aporia_potanini_potanini_2020POT03_OR883514_China_Shaanxi_Xian
Aporia_potanini_intercostata_NC_025273_AND_KC461928_Genbank
Aporia_potanini_huanghaoi_2022PTN02_OR178658_China_Beijing_Mentougou
Aporia_potanini_APOT-CHPBE3_China_Beijing
Aporia_potanini_APOT-CHPBE1_China_Beijing
Aporia_potanini_APOT-CHPBE2_China_Beijing
Aporia_peloria_M.pell_KU921272_China_Gansu_Xiahe
Aporia_peloria_MPEL-CHQIA1_China_Tibet
Aporia_peloria_MPEL-CHLAS1_China_Tibet
Aporia_peloria_MP-2011-57CI_JQ922062_China_Qinghai_Huzhu
Aporia_peloria_NC_033891_AND_KX495168_China_Qinghai_Qilian_Mts
Aporia_peloria_mp2_OR491857_Genbank
Aporia_peloria_mp1_OR491853_Genbank
Aporia_peloria_mp3_OR491861_Genbank
Aporia_martineti_martineti_2022MAR07_OQ992592_China_Yunnan_Diqing
Aporia_martineti_martineti_AMAR-CHYZS1_China_Yunnan
Aporia_martineti_konbogyandaensis_AMAR-CHTML1_China_Tibet
Aporia_martineti_martineti_2022MAR06_OR178651_China_Gansu_Lanzhou
Aporia_martineti_martineti_2022MAR04_OQ992591_China_Yunnan_Lijiang
Aporia_martineti_am1_OR491850_Genbank
Aporia_martineti_am3_OR491860_Genbank
Aporia_martineti_am2_OR491855_Genbank
Aporia_martineti_NC_033890_AND_KX495167_China_Gansu
Aporia_bieti_lihsieni_ABIE-CHSYL1_China_Sichuan
Aporia_bieti_lihsieni_ABIE-CHSYL2_China_Sichuan
Aporia_bieti_xizangensis_ABIE-CHTNV1_China_Tibet
Aporia_bieti_xizangensis_ABIE-CHTRL1_China_Tibet
Aporia_bieti_OP526834_Genbank
Aporia_bieti_misidentified_as_hippia_AHIP-CHSYS1_China_Sichuan
Aporia_bieti_gregoryi_2022BIT01_OQ992572_China_Yunnan_Lijiang
Aporia_bieti_gregoryi_2022BIT08_OQ992573_China_Yunnan_Diqing
Aporia_bieti_HJS-200601_EF584850_China_Yunnan
Aporia_bieti_A.bie1_KU921256_China_Shaanxi_Fengxian
Aporia_hippia_ChiEu3-175-12_LC471697_Russia_Khabarovsky_Amur_region
Aporia_hippia_Eu4-255-92_Russia_Khabarovskyi_Amur
Aporia_hippia_ChiJp1-64-12_LC471698_Japan_Nagano_Yatsugatake_Mts
Aporia_hippia_NhiJp1-64-13_Japan_Yamanashi_Yatsugatake_Mts
Aporia_hippia_japonica_AB107922_Japan_Nagano
Aporia_hippia_ChiEu1-175-11_LC471695_Mongolia_Tov_Terelji
Aporia_hippia_ChiEu2-245-2_LC471696_China_Beijing
Aporia_hippia_japonica_HaplotypeA_LC639998_Japan_Honshu
Aporia_hippia_japonica_HaplotypeB_LC639999_Japan_Honshu
Aporia_hippia_misidentified_as_bieti_NC_033888_AND_KX495165_China_Gansu
Aporia_hippia_AH-2007-07CI_JQ922053_China_Qinghai_Huzhu
Aporia_hippia_A.hip1_KU921262_China_Shaanxi_Taochuan
Aporia_hippia_taupingi_2022HIP02_OR178647_China_Gansu_Lanzhou
Aporia_hippia_cratagegioides_2022HIP01_OR178646_China_Beijing_Mentougou
Aporia_hippia_misidentified_as_bieti_ab1_OR491810_Genbank
Aporia_hippa_misidentified_as_bieti_ab2_OR491811_Genbank
Aporia_signiana_ASIG-CHSRW1_China_Sichuan
Aporia_tsinglingica_2022TSL02_OR178660_China_Shaanxi_Baoji
Aporia_tsinglingica_2022TSL01_OR178659_China_Shaanxi_Hanzhong
Aporia_tsinglingica_ATSI-CHQSH1_China_Shaanxi_Qingling_Shan
Aporia_procris_sinensis_2018PRO05_OR883522_China_Qinghai
Aporia_procris_sinensis_2018PRO06_OR883523_China_Qinghai_Haidong
Aporia_procris_procris_APRO-CHSWB1_China_Sichuan
Aporia_procris_procris_APRO-CHSKN2_China_Sichuan
Aporia_uedai_2022UED01_OQ992604_China_Yunnan_Diqing
Aporia_uedai_AUED-CHYDE1_China_Yunnan
Aporia_lhamo_ALHA-CHYDE2_China_Yunnan
Aporia_lhamo_2022LAM03_OQ992590_China_Yunnan_Diqing
Aporia_lhamo_2022LAM01_OQ992589_China_Yunnan_Diqing
Aporia_lhamo_chunhaoi_2020CHU01_OR178666_China_Yunnan_Lijiang
Aporia_lhamo_chunhaoi_2020CHU02_OR178667_China_Yunnan_Lijiang
Aporia_procris_nyanchuensis_APRO-CHTNV1_China_Tibet
Aporia_procris_nyanchuensis_APRO-CHTNV2_China_Tibet
Aporia_procris_procris_APRO-CHSKN1_China_Sichuan
Aporia_procris_lancangica_APRO-CHYMV1_China_Yunnan
Aporia_procris_APRO-CHYZS1_China_Yunnan
Aporia_procris_lancangica_2022PRO04_OQ992601_China_Yunnan_Diqing
Aporia_procris_procris_2022PRO03_OQ992603_China_Yunnan_Lijiang
Aporia_procris_APRO-CHYNR2_China_Yunnan
Aporia_procris_procris_2022PRO02_OQ992602_China_Yunnan_Lijiang
Aporia_belucha_belucha_SMNS-VN10_Pakistan_Baluchistan
Aporia_belucha_belucha_SMNS-VN11_Pakistan_Baluchistan
Aporia_belucha_belucha_SMNS-VN02_Pakistan_Baluchistan
Aporia_belucha_leechi_DNAwthAporia008_Afghanistan_Panjshir
Aporia_belucha_leechi_DDB-VN07_Afghanistan_Panjshir
Aporia_belucha_leechi_ZFMK-LepDNA0000165_Pakistan_Chitral
Aporia_nabellica_ANAB-PKKVS1_Pakistan_Kaghan_Valley
Aporia_nabellica_ANAB-PKKVS2_Pakistan_Kaghan_Valley
Aporia_soracta_sara_ALEU-NPDOD3_Nepal_Dolpo_Likhu
Aporia_soracta_sara_ALEU-NPDOD1_Nepal_Dolpo_Likhu
Aporia_soracta_sara_ALEU-NPDOD2_Nepal_Dolpo_Likhu
Aporia_soracta_soracta_DNAcdb0129_India_Uttarakhand
Aporia_soracta_soracta_DNAcdb0128_India_Himachal_Pradesh
Aporia_soracta_soracta_AF-VN08_India_Himachal_Pradesh
Aporia_soracta_soracta_GCB-Ap7_India_Himachal_Pradesh
Aporia_ahura_sp_nov._ARPI-9999-029_MN993007_Iran_Tehran_Dizin
Aporia_ahura_sp_nov._ARPI-9999-031_MN993000_Iran_Tehran_Dizin
Aporia_ahura_sp_nov._ARPI-9999-032_MN993017_Iran_Khorasan_Bojnurd_mislabeled
Aporia_ahura_sp_nov._ARPI-9999-030_MN993029_Iran_Tehran_Dizin
Aporia_illumina_DNAwthAporia017_Iran_Semnan_Shahkuh
Aporia_illumina_DNAwthAporia010_Iran_Khorasan-e_Jonubi_Kuh-e_Sorkh
Aporia_illumina_DNAwthAporia018_Iran_Mazandaran_Kuh-e_Shavar
Aporia_illumina_DNAwthAporia011_Iran_Khorasan-e_Jonubi_Kuh-e_Sorkh
Aporia_illumina_DNAwthAporia014_Iran_Mazandaran_Kuh-e_Shavar
Aporia_illumina_DNAwthAporia012_Iran_Khorasan-e_Jonubi_Kuh-e_Sorkh
Aporia_illumina_ZFMK-LepDNA0000169_Iran_Semnan_Shahkuh
Aporia_illumina_RPVL-00266_Iran_Shahkuh
Aporia_illumina_DNAwthAporia015_Iran_Mazandaran_Kuh-e_Shavar
Aporia_illumina_DNAwthAporia016_Iran_Golestan_Shahkuh
Aporia_illumina_DNAwthAporia013_Iran_Semnan_Shahkuh
Aporia_illumina_DNAwthAporia009_Iran_Khorasan-e_Jonubi_Kuh-e_Sorkh
Aporia_illumina_ZFMK-LepDNA0000170_Iran_Semnan_Shahkuh
Aporia_leucodice_leucodice_GCB-Ap2_Kazakhstan_Almaty
Aporia_leucodice_leucodice_GCB-Ap1_Kyrgyzstan_Batken
Aporia_leucodice_aryana_DNAcdb0121_Afghanistan_Panjshir_valley
Aporia_leucodice_aryana_GCB-Ap3_Afghanistan_Panjshir
Aporia_leucodice_leucodice_ALEU-KZTDO1_Kyrgyzstan_Dolon_Pass
Aporia_leucodice_leucodice_DNAcdb0117_Tajikistan_Darvaz_Peter_I_range
Aporia_leucodice_leucodice_ALEU-KZDOP1_Tajikistan_Kunbel
Aporia_leucodice_morosevitschae_DNAwthAporia003_Uzbekistan
Aporia_leucodice_leucodice_DNAwthAporia005_Kyrgyzstan
Aporia_leucodice_leucodice_2005-LOWA-279_FJ663280_Kazakhstan_Dshungarski_Alatau
Aporia_leucodice_morosevitschae_DNAwthAporia004_Kazakhstan
Aporia_leucodice_leucodice_DNAcdb0120_Kyrgyzstan_TianShan
Aporia_leucodice_leucodice_2005-LOWA-353_FJ663278_Kyrgyzstan_Naryn
Aporia_leucodice_leucodice_2005-LOWA-277_FJ663282_Kazakhstan_Dshungarski_Alatau
Aporia_leucodice_leucodice_2005-LOWA-354_FJ663277_Kyrgyzstan_Naryn
Aporia_leucodice_leucodice_2005-LOWA-278_FJ663281_Kazakhstan_Dshungarski_Alatau
Aporia_leucodice_leucodice_A.leu1_KU921264_China_Xinjiang_Qinghe
Aporia_leucodice_leucodice_DNAwthAporia001_Kyrgyzstan
Aporia_leucodice_leucodice_SMNS-VN12_China_Xinjiang
Aporia_leucodice_leucodice_DNAwthAporia006_Kyrgyzstan
Aporia_leucodice_leucodice_SMNS-VN11_China_Xinjiang
Aporia_leucodice_leucodice_2005-LOWA-280_FJ663279_Kazakhstan_Dshungarski_Alatau
Aporia_leucodice_leucodice_DNAwthAporia007_Kyrgyzstan
Aporia_delavayi_minschani_2022DEL05_OR178642_China_Shaanxi_Baoji
Aporia_delavayi_minschani_2022DEL06_OR178643_China_Shaanxi_Baoji
Aporia_delavayi_delavayi_A.del1_KU921258_AND_KM669581_China_Shaanxi_Huoditang
Aporia_delavayi_2022DEL01_OQ992576_China_Shaanxi_Huoditang
Aporia_delavayi_delavayi_2022DEL02_OQ992577_China_Yunnan_Diqing
Aporia_delavayi_dayensis_ADEL-CHSDH1_China_Sichuan
Aporia_delavayi_ADEL-CHSWB3_China_Sichuan
Aporia_delavayi_ADEL-CHSWB1_China_Sichuan
Aporia_delavayi_ADEL-CHSWB2_China_Sichuan
Aporia_goutellei_misidentified_as_acraea_isolate_APA_China_Sichuan_Yaan
Aporia_goutellei_AGOU-CHSWE1_China_Sichuan
Aporia_goutellei_OQ992579_China_Yunnan_Lijiang
Aporia_goutellei_goutellei_2022GOT04_OQ992580_China_Yunnan_Lijiang
Aporia_howarthi_AHOR-CHTGY1_China_Tibet
Aporia_goutellei_A.gou1_KU921261_China_Shaanxi_Huoditang
Aporia_goutellei_goutellei_2011GOT15_OR883525_China_Shaanxi_Ankang
Aporia_goutellei_goutellei_2015GOT14_OR883524_China_Shaanxi_Ankang
Aporia_goutellei_nitida_AGOU-CHSCH1_China_Sichuan
Aporia_goutellei_xantina_AGOU-CHECMV2_China_Tibet
Aporia_kanekoi_AKAN-CHSDY1_China_Sichuan
Aporia_kanekoi_AKAN-CHSWD1_China_Sichuan
Aporia_melania_nutans_2022MEL04_OQ992593_China_Yunnan_Lijiang
Aporia_melania_nutans_2022MEL05_OQ992594_China_Yunnan_Diqing
Aporia_larraldei_melanica_ALAR-CHSKR3_China_Sichuan
Aporia_larraldei_shizouyai_ALAR-CHSWD1_China_Sichuan
Aporia_larraldei_ALAR-CHSKR1_China_Sichuan
Aporia_larraldei_larraldei_ALAR-CHSKR2_China_Sichuan
Aporia_agathon_moltrechti_AAGA-TWPUL2_Taiwan_Puli
Aporia_agathon_moltrechti_MY-2016-6_ON436234_Taiwan_Taichung
Aporia_agathon_bifurcata_2022AGT01_OQ992574_China_Yunnan_Kunming
Aporia_agathon_bifurcata_2022AGT02_OQ992575_China_Yunnan_Dali
Aporia_largeteaui_A.lar1_KU921263_China_Shaanxi_Fengxian
Aporia_largeteaui_misidentified_as_gigantea_A.gig1_KU921260_China_Sichuan_Emeishan
Aporia_largeteaui_NC_058011_AND_MW284889_China
Aporia_agathon_AAGA-BTKYO1_Bhutan
Aporia_agathon_AAGA-BTTBC1_Bhutan
Aporia_agathon_misidentified_as_harrietae_AHAR-BTWDN1_Bhutan
Aporia_agathon_misidentified_as_harrietae_AHAR-BTWDN2_Bhutan
Aporia_agathon_AAGA-NPDDL1_Nepal_Western
Aporia_agathon_AAGA-NPDDL2_Nepal_Western
Aporia_agathon_A-11856A_KU640959_India_Uttarakhand_Mussorie
Aporia_agathon_A-11856B_KU640960_India_Uttarakhand_Mussorie
Aporia_agathon_A-11758_KU640962_India_Uttarakhand_Mussorie
Aporia_agathon_A-11759_KU640963_India_Uttarakhand_Mussorie
Aporia_agathon_A-11757_KU640961_India_Uttarakhand_Mussorie
Aporia_agathon_phryxe_AAGA-INDKCH1_India_Himachal_Pradesh
Aporia_agathon_A-11760_KU640964_India_Uttarakhand_Mussorie
Aporia_agathon_phryxe_AAGA-INDHPR1_India_Himachal_Pradesh
Aporia_agathon_AAGA-NPKPU3_Nepal_Kathmandu
Aporia_agathon_agathon_AAGA-NPKVN1_Nepal_Kathmandu
Aporia_agathon_AAGA-NPKVP2_Nepal_Kathmandu
Aporia_agathon_AAGA-NPMPT1_Nepal_Makwanpur
Aporia_agathon_AAGA-CHTNT1_China_Tibet
Aporia_agathon_AAGA-CHTNT2_China_Tibet
Aporia_agathon_omotoi_2022AGT05_OQ992600_China_Yunnan_Diqing
Aporia_agathon_gaolingonshanensis_2019AGT11_OQ992597_China_Yunnan_Nujiang
Aporia_agathon_gaolingonshanensis_2019AGT12_OQ992598_China_Yunnan_Nujiang
Aporia_agathon_omotoi_2022AGT03_OQ992599_China_Yunnan_Diqing
Aporia_agathon_omotoi_AAGA-CHYSV1_China_Yunnan
Aporia_agathon_gaolingonshanensis_2023AGT14_OR965373_China_Yunnan_Dehong
Aporia_agathon_gaolingonshanensis_2023AGT16_OR965375_China_Yunnan_Dehong
Aporia_agathon_gaolingonshanensis_2023AGT15_OR965374_China_Yunnan_Dehong
Aporia_agathon_gaolingonshanensis_2023AGT13_OR965372_China_Yunnan_Dehong
Aporia_agathon_gaolingonshanensis_2023AGT17_OR965376_China_Yunnan_Dehong
Aporia_agathon_MFB-00-P481_DQ082760_China_Yunnan_nr_Lijang
Aporia_agathon_A.aga1_KU921255_AND_KM669578_Tibet_Motuo
Aporia_agathon_KU921255
Aporia_agathon_omotoi_AAGA-CHYSB1_China_Yunnan
Aporia_agathon_omotoi_AAGA-CHYSB2_China_Yunnan
Aporia_gigantea_ALRG-CHSKL1_China_Sichuan
Aporia_gigantea_misidentified_as_intercostata_HJS-200602_EF584851_China_Anhui
Aporia_gigantea_AGIG-CHSDA1_China_Sichuan
Aporia_gigantea_AGIG-CHSDD1_China_Sichuan
Aporia_gigantea_AGIG-CHSDS1_China_Sichuan
Aporia_largeteaui_largeteaui_2022LAG03_OR178648_China_Hubei_Xiangyang
Aporia_largeteaui_largeteaui_2022LAG04_OR178649_China_Hubei_Xiangyang
Aporia_largeteaui_largeteaui_2022LAG05_OR178650_China_Guizhou_Tongren
Aporia_lemoulti_ALEM-CHSSD1_China_Sichuan
Aporia_lemoulti_ALEM-CHSDP3_China_Sichuan
Aporia_hastata_2022HAS04_OQ992584_China_Yunnan_Dali
Aporia_hastata_NC_068354_AND_OP373108_China_Yunnan
Aporia_hastata_2022HAS03_OQ992583_China_Yunnan_Dali
Aporia_hastata_AHAS-CHYLA1_China_Yunnan
Aporia_hastata_AHAS-CHYLA2_China_Yunnan
Aporia_bernardi_yunnanica_2022BER01_OQ992570_China_Yunnan_Lijiang
Aporia_bernardi_yunnanica_2022BER08_OQ992571_China_Yunnan_Lijiang
Aporia_bernardi_bernardi_ABER-CHSLB1_China_Sichuan
Aporia_bernardi_ABER-CHGSM2_China_Ghizhou
Aporia_bernardi_yunnana_ABER-CHYDE2_China_Yunnan
Aporia_kamei_2022KAM10_OQ992587_China_Yunnan_Diqing
Aporia_kamei_2022KAM12_OQ992588_China_Yunnan_Diqing
Aporia_kamei_AKAM-CHYLZ1_China_Yunnan
Aporia_kamei_2022KAM02_OQ992586_China_Yunnan_Diqing
Aporia_kamei_2022KAM01_OQ992585_China_Yunnan_Diqing
Aporia_kamei_AKAM-CHYNR2_China_Yunnan
Aporia_oberthuri_cinerea_AOBE-CHSWD1_China_Sichuan
Aporia_oberthuri_cinerea_AOBE-CHSDU1_China_Sichuan
Aporia_oberthuri_A.obe1_KU921266_China_Shaanxi_Huoditang
Aporia_oberthuri_AOB-CHSHTH1_China_Shaanxi
Aporia_oberthuri_AOBE-CHSHX1_China_Shaanxi
Aporia_oberthuri_AOBE-CHQSX1_China_Shaanxi_Qingling_Shan
Aporia_oberthuri_cinerea_2020OBT01_OR178655_China_Chongqing_Wuxi
Aporia_oberthuri_cinerea_2020OBT02_OR178656_China_Chongqing_Wuxi
Aporia_oberthuri_AOBE-CHSHQ2_China_Shaanxi
Aporia_oberthuri_oberthuri_2020OBT05_OR883520_China_Shaanxi_Ankang
Aporia_oberthuri_oberthuri_2019OBT06_OR883521_China_Shaanxi_Hanzhong
Aporia_oberthuri_oberthuri_2021OBT04_OR883519_China_Shaanxi_Xian
Aporia_acraea_2022ACR01_OQ992569_China_Yunnan_Diqing
Aporia_acraea_wolongensis_koiwayai_2022WOL01_OQ992605_China_Yunnan_Lijiang
Aporia_acraea_wolongensis_koiwayai_2022WOL05_OQ992606_China_Yunnan_Diqing
Aporia_acraea_acraea_AACR-CHSWB1_China_Sichuan
Aporia_acraea_wolongensis_misidentified_as_goutellei_ON533767_China_Sichuan
Aporia_acraea_wolongensis_AACR-CHSWB2_China_Sichuan
Aporia_acraea_wolongensis_AACR-CHSWB3_China_Sichuan
Aporia_monbeigi_meiliiensis_AMON-CHYMV1_China_Yunnan
Aporia_monbeigi_2022MOB01_OQ992595_China_Yunnan_Diqing
Aporia_monbeigi_2022MOB02_OQ992596_China_Yunnan_Diqing
Aporia_monbeigi_meiliiensis_AMON-CHYME1_China_Yunnan
Aporia_monbeigi_monbeigi_AMON-CHYWX1_China_Yunnan
Aporia_monbeigi_monbeigi_AMON-CHYWX2_China_Yunnan
Aporia_nishimurai_shinnooka_2022NIS06_OR178654_China_Chongqing_Wuxi
Aporia_tayiensis_siyaoi_2022TAY02_OR178665_China_Gansu_Longnan
Aporia_tayiensis_siyaoi_2022TAY01_OR178664_China_Gansu_Longnan
Aporia_nishimurai_ANIS-CHSSS1_China_Sichuan
Aporia_nishimurai_nishimurai_2022NIS03_OR178653_China_Sichuan_Leshan
Aporia_nishimurai_nishimurai_2022NIS02_OR178652_China_Sichuan_Leshan
Aporia_tayensis_ATAY-CHSDD1_China_Sichuan
Aporia_harrietae_paracraea_2022HAR03_OQ992581_China_Yunnan_Lijiang
Aporia_harrietae_paracraea_AHAR-CHYNS1_China_Yunnan
Aporia_harrietae_paracraea_2023HAR01_OR883511_China_Yunnan_Nujiang
Aporia_harrietae_paracraea_2023HAR02_OR883512_China_Yunnan_Nujiang
Aporia_harrietae_AHAR-BUKAK2_Bhutan
Aporia_harrietae_AHAR-BUKAK3_Bhutan
Aporia_harrietae_AHAR-BUKAK1_Bhutan
Aporia_harrietae_AHAR-BUKAK4_Bhutan
Aporia_harrietae_paracraea_2023HAR04_OR883513_China_Yunnan_Nujiang
Aporia_joubini_NHMUK010201224_MN993055_China_Yunnan_Tsekou
Aporia_harrietae_paracraea_2022HAR04_OQ992582_China_Yunnan_Diqing
Aporia_harrietae_paracraea_AHAR-CHYTK1_China_Yunnan
Aporia_harrietae_AHAR-BTKYO1_Bhutan
Aporia_harrietae_AHAR-BTTYW1_Bhutan
Aporia_harrietae_AHAR-BTTDT1_Bhutan
Aporia_harrietae_AHAR-BTTDT2_Bhutan
Aporia_harrietae_baylei_AHAR-CHTNP1_China_Tibet
Aporia_harrietae_chiara_AHAR-BTJDH1_Bhutan
Aporia_harrietae_misidentified_as_monbeigi_AMON-BTJDH1_Bhutan
Aporia_harrietae_AHAR-BTTDT3_Bhutan
Aporia_harrietae_harrietae_AHAR-BTTDT6_Bhutan
Aporia_harrietae_AHAR-BTTDH2_Bhutan
Aporia_harrietae_AHAR-BTTDH3_Bhutan
Aporia_harrietae_AHAR-BTTDH1_Bhutan
Aporia_harrietae_AHAR-BTTDT4_Bhutan
Aporia_harrietae_AHAR-BTTDT5_Bhutan
0.03

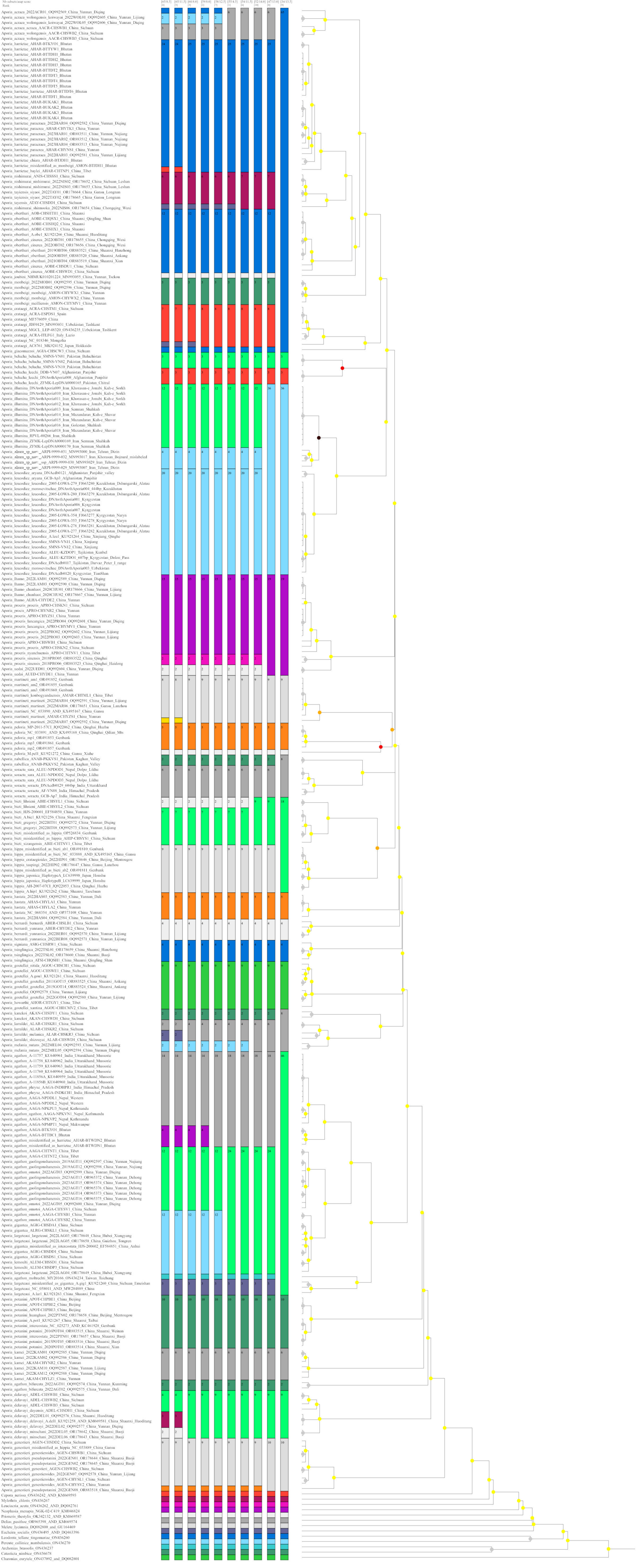

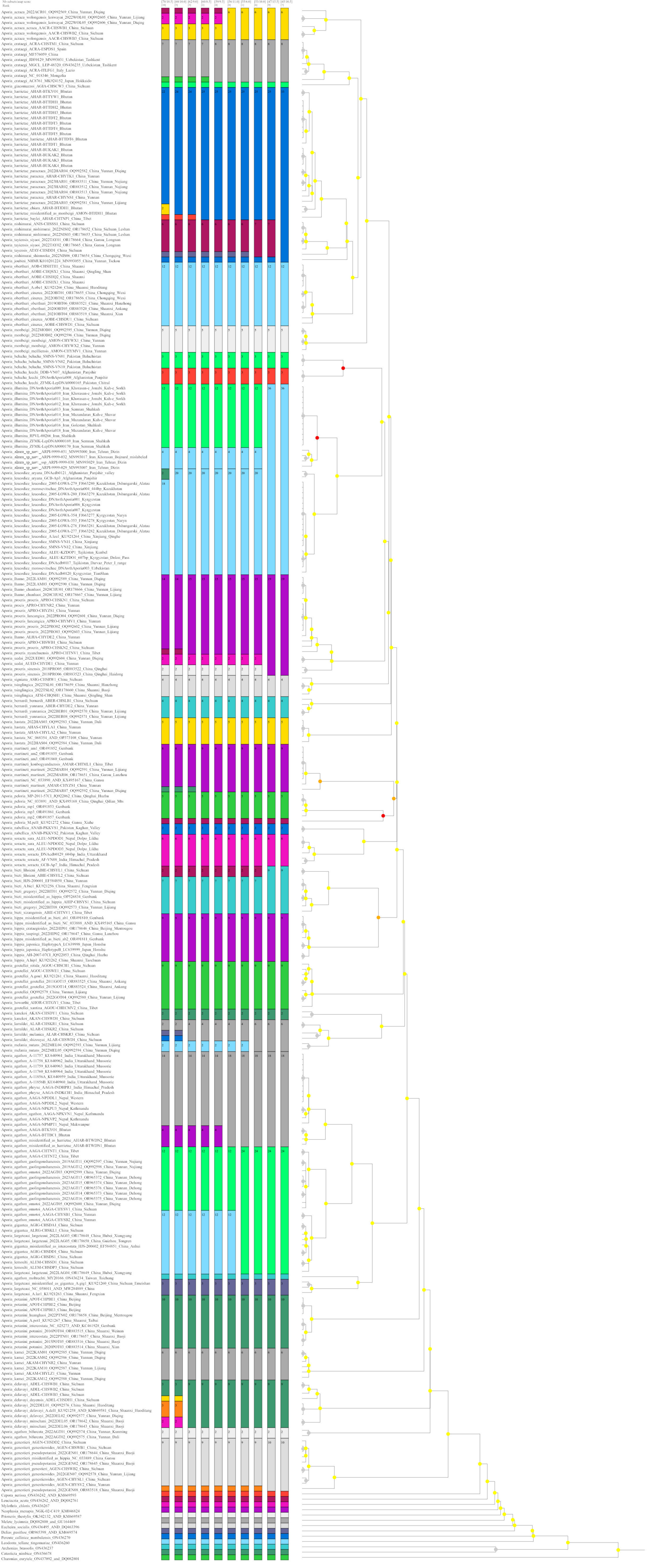

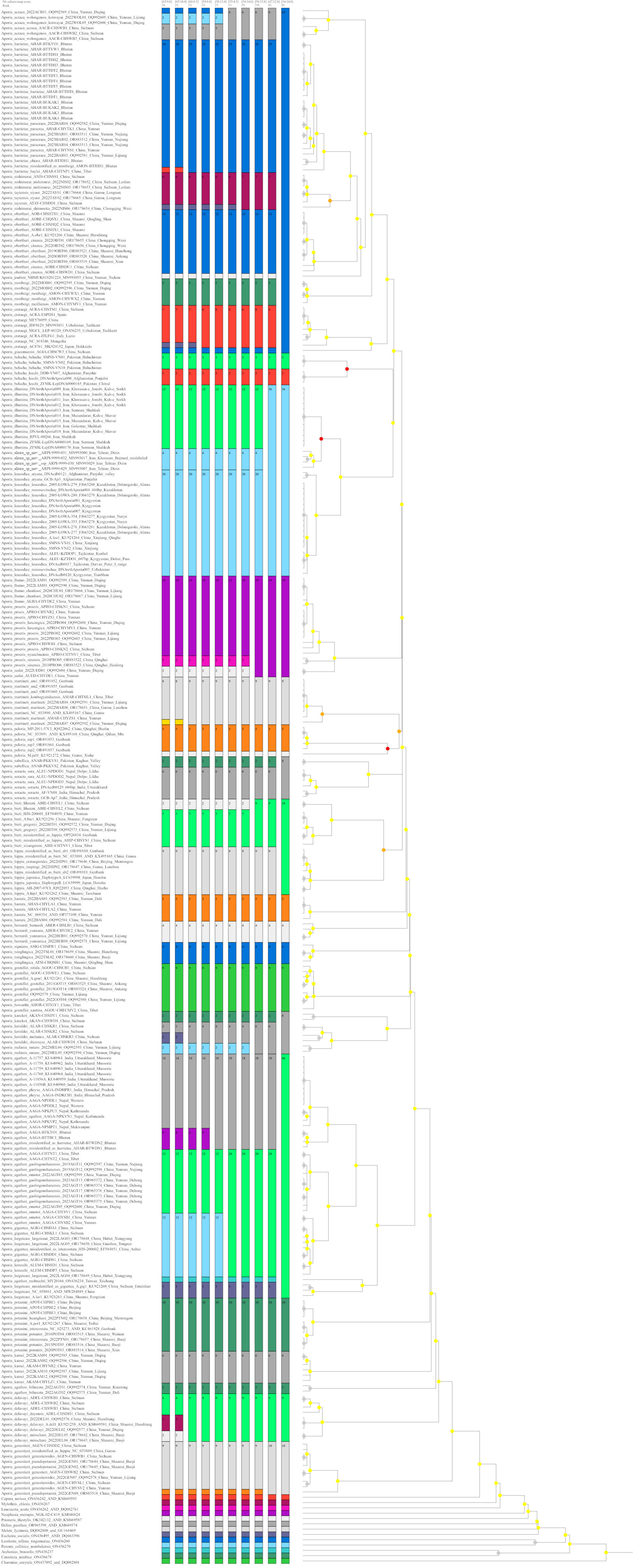

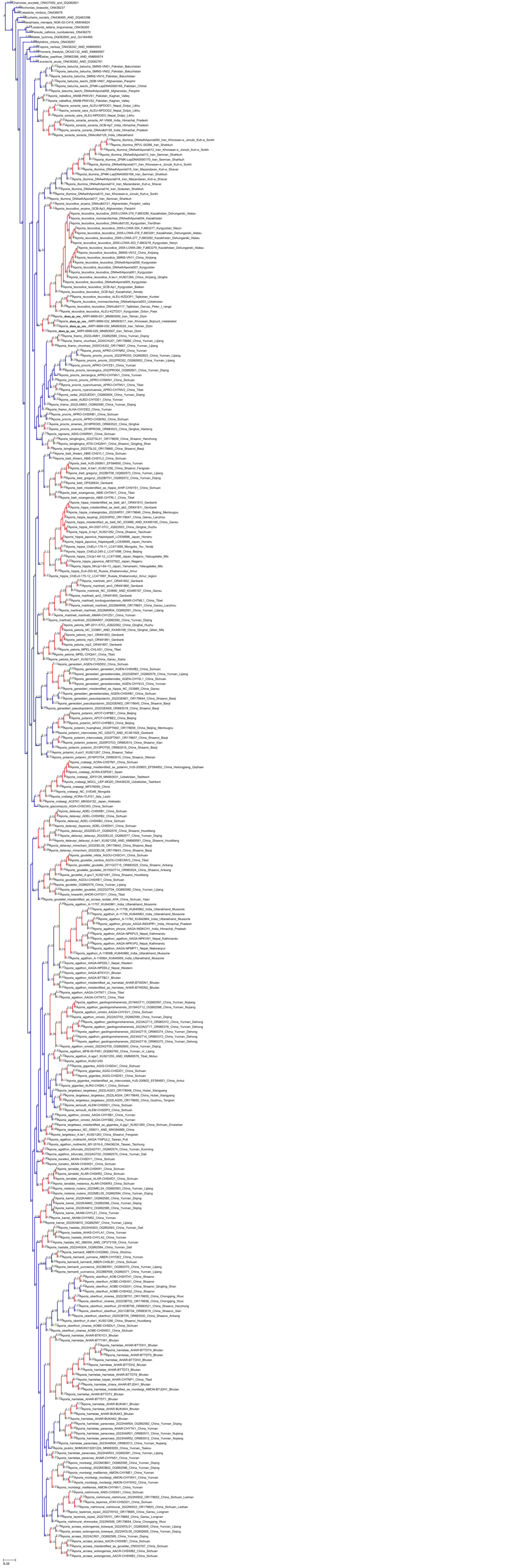

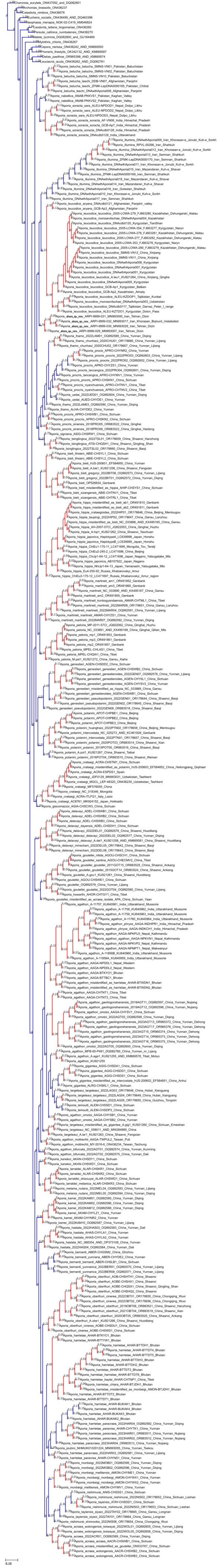

Model	LnL	n. params	d	e	j	AICc	AICc_wt
DEC	-327.8	2	0.0036	1.0e-12	0	659.7	1.5e-19
**DEC+J**	**-284.1**	**3**	**0.0002**	**1.0e-12**	**0.012**	**574.3**	**0.53**
DIVALIKE	-342.8	2	0.0052	1.0e-12	0	689.6	4.8e-26
DIVALIKE+J	-284.2	3	0.0002	1.0e-12	0.012	574.6	0.46
BAYAREALIKE	-461.7	2	0.0028	0.083	0	927.4	1.1e-77
BAYAREALIKE+J	-288.3	3	0.0001	1.0e-07	0.012	582.7	0.0080

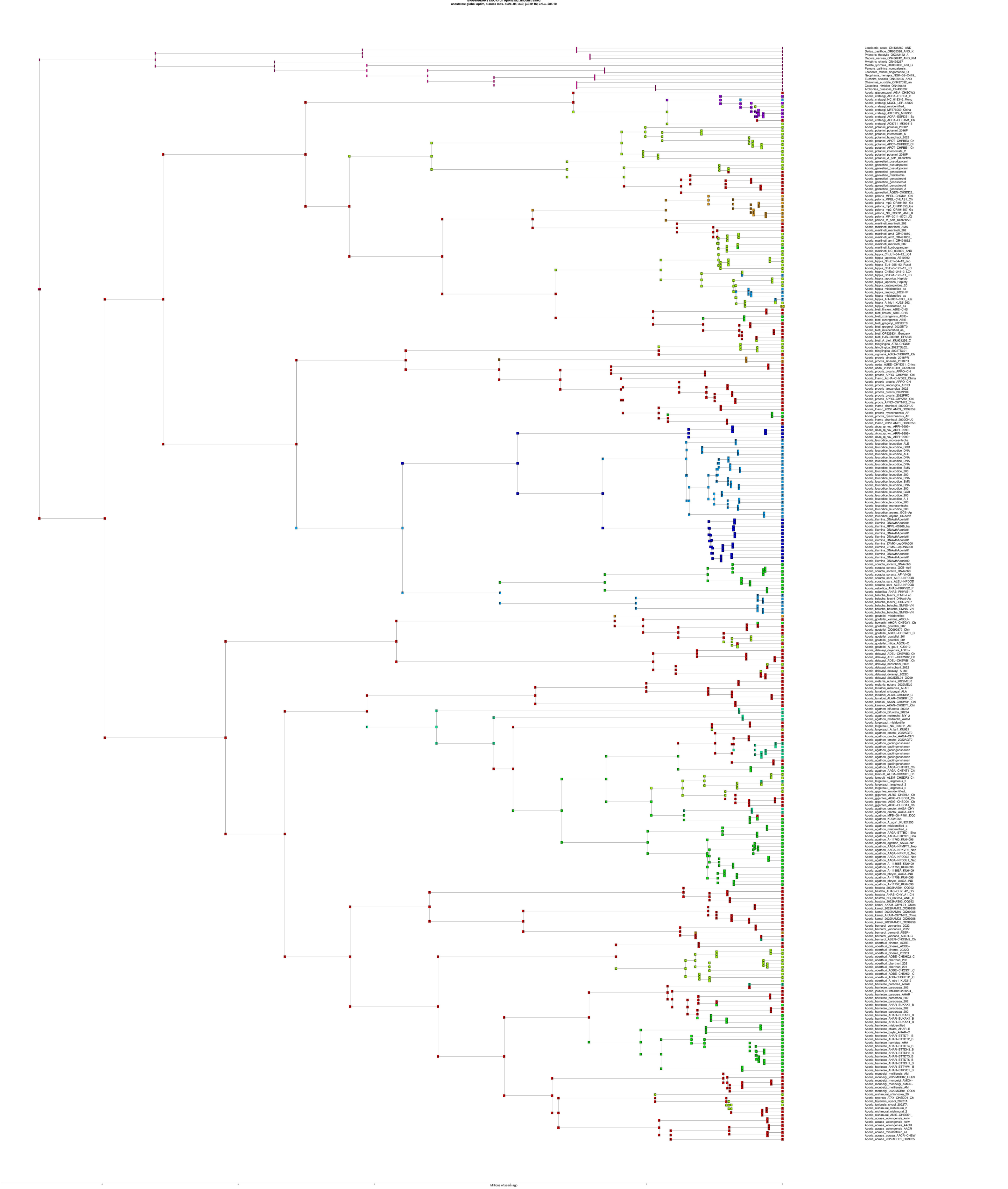
BioGeoBEARS DEC+J on Aporia M0_unconstrained
ancstates: global optim, 4 areas max. d=2e−04; e=0; j=0.0116; LnL=−284.10
Millions of years ago

BioGeoBEARS DEC+J on Aporia M0_unconstrained
ancstates: global optim, 4 areas max. d=2e−04; e=0; j=0.0116; LnL=−284.10

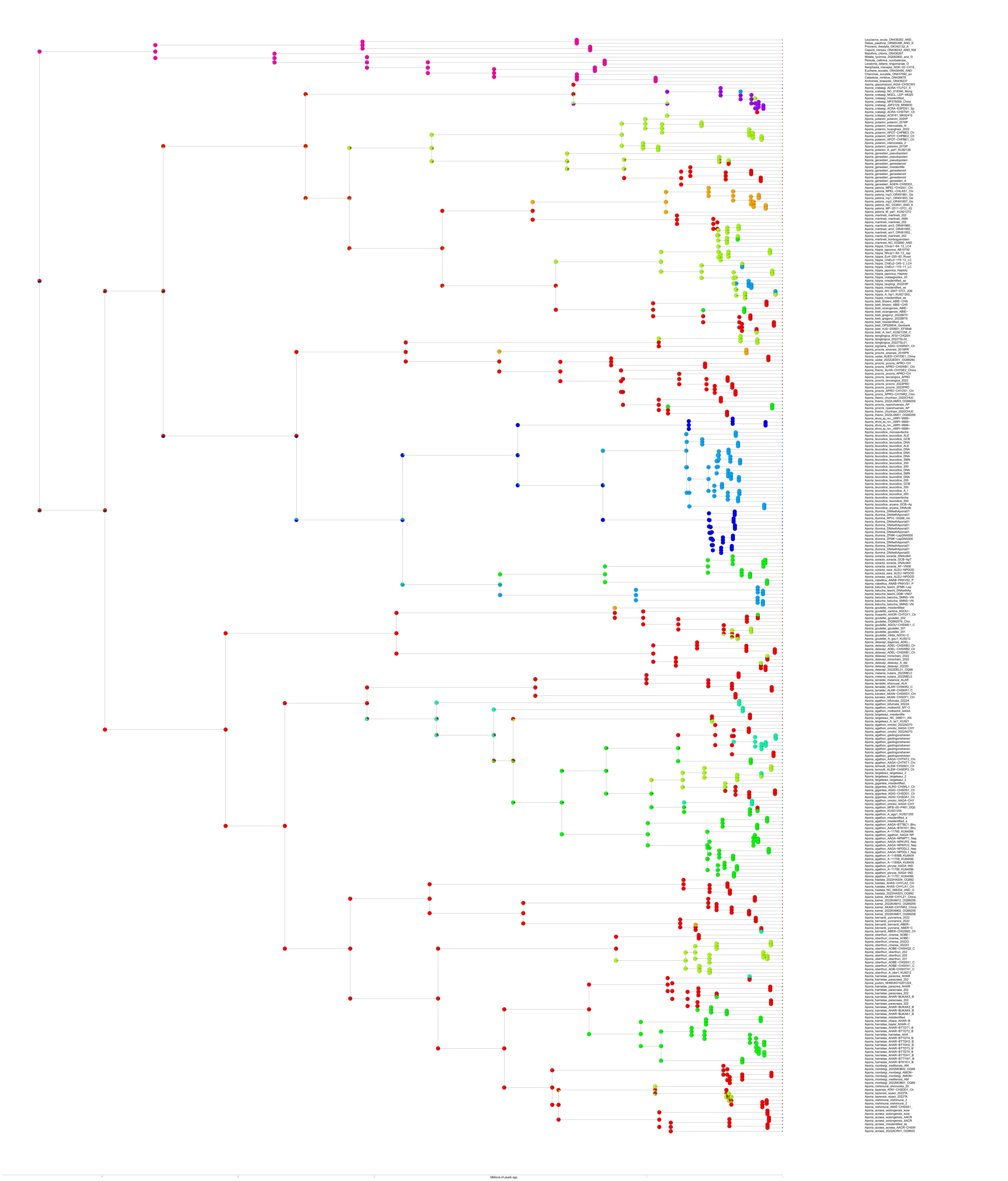

Supplement: Supplementary file 2 — Supplementary Material 2. [file 12862_2024_2294_MOESM2_ESM.pdf]
